# Promoting Singlet/triplet Exciton Transformation in Organic Optoelectronic Molecules: Role of Excited State Transition Configuration

**DOI:** 10.1038/s41598-017-05339-4

**Published:** 2017-07-24

**Authors:** Runfeng Chen, Yuting Tang, Yifang Wan, Ting Chen, Chao Zheng, Yuanyuan Qi, Yuanfang Cheng, Wei Huang

**Affiliations:** 1 Key Laboratory for Organic Electronics and Information Displays & Institute of Advanced Materials, Jiangsu National Synergetic Innovation Center for Advanced Materials, Nanjing University of Posts and Telecommunications, Wenyuan Road, Nanjing, 210023 P.R. China; 2 Key Laboratory of Flexible Electronics & Institute of Advanced Materials, National Synergetic Innovation Center for Advanced Materials, Nanjing Tech University, 30 South Puzhu Road, Nanjing, 211816 P.R. China

## Abstract

Exciton transformation, a non-radiative process in changing the spin multiplicity of an exciton usually between singlet and triplet forms, has received much attention recently due to its crucial effects in manipulating optoelectronic properties for various applications. However, current understanding of exciton transformation mechanism does not extend far beyond a thermal equilibrium of two states with different multiplicity and it is a significant challenge to probe what exactly control the transformation between the highly active excited states. Here, based on the recent developments of three types of purely organic molecules capable of efficient spin-flipping, we perform *ab initio* structure/energy optimization and similarity/overlap extent analysis to theoretically explore the critical factors in controlling the transformation process of the excited states. The results suggest that the states having close energy levels and similar exciton characteristics with same transition configurations and high heteroatom participation are prone to facilitating exciton transformation. A basic guideline towards the molecular design of purely organic materials with facile exciton transformation ability is also proposed. Our discovery highlights systematically the critical importance of vertical transition configuration of excited states in promoting the singlet/triplet exciton transformation, making a key step forward in excited state tuning of purely organic optoelectronic materials.

## Introduction

**Figure 1:**
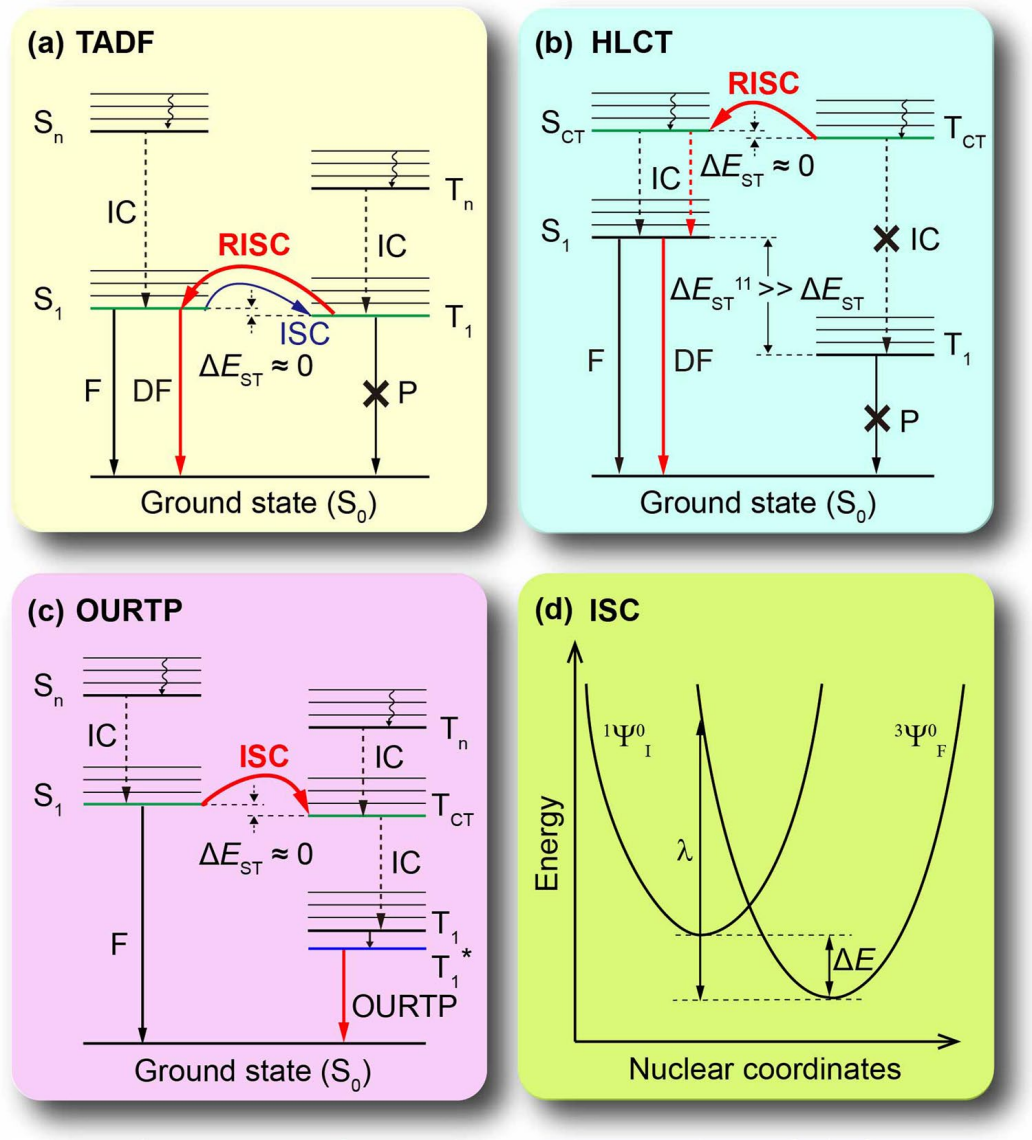
Energy level diagrams depicting diversified excited states transfer for exciton transformation in (**a**) TADF, (**b**) HLCT and (**c**) OURTP molecules and (**d**) harmonic energy surfaces representing ISC process. Noted that F, DF, P, IC represent fluorescence, delayed fluorescence, phosphorescence and internal conversion, respectively; Δ*E*_ST_ is the energy splitting between the singlet states and triplet states, while Δ*E*_ST_^11^ is the energy splitting between the lowest singlet (S_1_) and triplet (T_1_) excited states.

When an organic molecule is excited to form a singlet or triplet exciton, the spin multiplicity of the exciton can transform either from singlet to triplet *via* intersystem crossing (ISC) or from triplet to singlet *via* reverse intersystem crossing (RISC)^1–3^. This phenomenon of exciton transformation has been known for decades, but important details of its photophysical mechanism remain unrevealed, let alone the rational molecular design for controlled exciton transformation between the highly active excited states. Nevertheless, the interest in exciton transformation of purely organic molecules has been greatly renewed in recent years, because of the significant effects in promoting device performance by properly adjusting excited states for the desired forms of excitons. For example, highly luminescent singlet excitons are favorable for organic light emitting diodes (OLEDs)^4, 5^, while long-lived triplet excitons are attractive for organic photovoltaics (OPVs) with long diffusion length to enhance exciton dissociation to generate free charges^6, 7^ and for lifetime-resolved encryption and sensor applications with long-lived phosphorescence to eliminate the disturbance of short-lived background fluorescence^8, 9^. Typically, by converting the electronically excited 75% triplet excitons to the singlet ones *via* RISC for thermally activated delayed fluorescence (TADF)^10^ or hybridized local charge-transfer (HLCT)^11^, a large amount of TADF (Fig. 1a) and HLCT (Fig. 1b) molecules for OLEDs have been prepared and their device performances are comparable to that of heavy metal-based complexes^12–14^. Meanwhile, exotic room-temperature phosphorescent (RTP) from purely organic materials has aroused much attention very recently with particular interests in luminescent mechanisms and molecule design principles of this novel kind of material^15, 16^. Especially, organic ultralong RTP (OURTP) molecules (Fig. 1c) has been found to exhibit ultralong phosphorescence with lifetime up to 1.35s upon photoexcitation under ambient conditions at room temperature^17^. In all these new-emerged purely organic materials of TADF, HLCT, and OURTP molecules, efficient exciton transformation has been experimentally confirmed and theoretically explained to be the origin of their extraordinary properties. These breakthroughs in facilitating spin-flipping are among the key issues and major achievements in the current development of organic electronics, posing strong demands on rational control of exciton transformation in purely organic molecules to take full advantages of different multiple forms of exciton with particular optical and electrical properties for desired applications.

The minimum requirement for realizing exciton transformation is the matching of the energy levels of the two states, based on a thermal equilibrium between the singlet and triplet excited states (energy gap law). Photophysical studies of TADF molecules with the small bandgap between the lowest singlet (S_1_) and triplet (T_1_) excited states offer an important path to understand the transformation mechanism. Both efficient ISC and RISC between S_1_ and T_1_ with rate constants up to ~10^6^ and 10^4^ s^−1^ respectively for exciton transformation have been experimentally identified in TADF materials and proved to be the key factor for their high OLED performance with external quantum efficiency (EQE) about 30%^18^. Another applicable guideline for enhancing ISC is El-Sayed rule^19^, which suggests that ^1^(*π*, π*) → ^3^(*n*, π*) ISC is faster than ^1^(*π*, π*) → ^3^(*π*, π*) transitions and ^1^(*n*, π*) → ^3^(*π*, π*) is faster than ^1^(*n*, π*) → ^3^(*n*, π*) transitions. This means the participation of heteroatom with either non-pair electrons or empty orbitals in the π-conjugated system is essential to promote the ISC process. Besides the energy gap law and El-Sayed rule, Golden Rule Approximation was also used to evaluate the exciton transformation. To facilitate the spin-flipping, the relativistic effect of spin orbital coupling (SOC) should be promoted through increased electromagnetic interaction between the electron’s spin and the magnetic field generated by the electron’s orbit around the nucleus^20^. The ISC rate (*k*_ISC_) from an initial singlet state (^1^ψ^0^_I_) to a final triplet state (^3^ψ^0^_F_) can be described in the golden-rule expression (Fig. 1d)^21, 22^:

1 $${k}_{{\rm{ISC}}}=\frac{1}{\hslash }{\langle {}^{{\rm{1}}}{\rm{\Psi }}_{{\rm{I}}}^{0}|{H}_{{\rm{SO}}}|{}^{{\rm{3}}}{\rm{\Psi }}_{{\rm{F}}}^{0}\rangle }^{{\rm{2}}}\sqrt{\frac{\pi }{\lambda RT}}\exp [-\frac{{({\rm{\Delta }}{\rm{E}}+\lambda )}^{2}}{4\lambda RT}]$$

where *λ* denotes the Marcus reorganization energy, ∆*E* is the energy difference between the initial and final states, and $\langle {}^{1}\Psi _{{\rm{I}}}^{0}|{H}_{S0}{|}^{3}{\Psi }_{F}^{0}\rangle$ is the expectation value of SOC. Using the first-order perturbation theory, the SOC interaction can be estimated by the zero-order eigenvectors^23^:

2 $$\begin{array}{c}\langle {}^{1}{\rm{\Psi }}^{0}|{H}_{{\rm{S0}}}|{}^{3}{\rm{\Psi }}^{0}\rangle ={\alpha }_{{\rm{fs}}}^{2}\sum _{\mu }^{{\rm{N}}}\sum _{{\rm{i}}}^{{\rm{n}}}\langle \frac{{Z}_{\mu }}{{r}_{{\rm{i}}\mu }^{3}}{}^{1}\psi ^{0}|\mathop{{L}_{{\rm{i}}}}\limits^{\longrightarrow}|{}^{3}\psi ^{0}\rangle \times \\ \langle \frac{1}{\sqrt{2}}(\alpha \beta -\beta \alpha )|\mathop{S}\limits^{\longrightarrow}|(\begin{array}{c}\alpha \alpha \\ \beta \beta \\ \frac{1}{\sqrt{2}}(\alpha \beta +\beta \alpha )\end{array})\rangle \end{array}$$

where *α*_fs_ is the fine structure constant, *Z*_*µ*_ is the effective nuclear charge for nucleus (*µ*), and *L* and *S* are the orbital and spin momenta, respectively. These calculations are rather complicated with considerable approximations, making the investigation of exciton transformation quite difficult, let alone giving a clear structure-property relation for rational control of both ISC and RISC in the molecular design of the related materials.

Here, we aim to investigate the crucial factors in determining the exciton transformation involved in three kinds of new-emerged purely organic optoelectronic materials of TADF, HLCT, and OURTP molecules capable of efficient exciton transformation. Density functional theory (DFT), time-dependent DFT (TD-DFT), and natural transition orbital (NTO) calculations^24, 25^ were performed to systematically study frontier orbital energy levels, overlap extents in both ground and excited states, and transition configuration similarity between the singlet/triplet excited states based on the ground state structure. With the newly proposed and computed quantitative parameters, we found that the two singlet and triplet excited states with close energy levels (±0.37 eV), similar exciton characteristics (containing the same transition configurations with high similarity) and high heteroatom participation are prone to exciton transformation for efficient ISC and RISC processes. This work can provide not only a facile theoretical method to evaluate the singlet/triplet exciton transformation, but also in-depth structure-property understandings into the exciton multiplicity manipulation.

## Results and Discussion

### Molecular Selection and Computational Methodology

**Figure 2:**
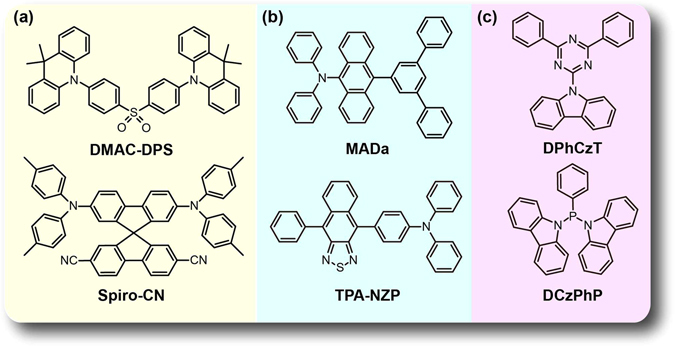
Chemical structures of typical (**a**) TADF (**DMAC-DPS** and **Spiro-CN**), (**b**) HLCT (**MADa** and **TPA-NZP**) and (**c**) OURTP (**DPhCzT** and **DCzPhP**) molecules.

Six typical purely organic molecules including two TADF (**DMAC-DPS** ^26^ and **Spiro-CN** ^27^), two HLCT (**MADa** ^28, 29^ and **TPA-NZP** ^29^), and two OURTP (**DPhCzT** and **DCzPhP** ^17^) molecules, which have been experimentally found to have efficient exciton transformation properties, were selected to investigate the exciton transformation principles at single molecular states (Fig. 2). It should be noted that all these molecules contain heteroatoms of N, S, O, or P to meet the requirement of El-Sayed rule in supporting the efficient exciton transformation. To choose a suitable calculation approach to investigate the highest occupied molecular orbital (HOMO), the lowest unoccupied molecular orbital (LUMO), vertical singlet (*E*_Sn_) and triplet (*E*_Tn_) excitation energies, and singlet–triplet energy splitting (Δ*E*_ST_) at excited states, we tested various DFT methods including B3LYP, PBE0, BMK, M06-2X, M06-HF that have different Hartree-Fock hybrid proportions, and long-range correction functional of *ω*B97XD at 6-31 G(d) basis set^30^. By comparing to the experimental results, the most applicable functionals for TADF, HLCT, and OURTP molecules are PBE0, M062X, and PBE0, respectively (Supplementary Table S1); they were then adopted in the following TD-DFT studies of the corresponding materials.

### Singlet-Triplet Splitting (Δ*E*_ST_)

**Figure 3:**
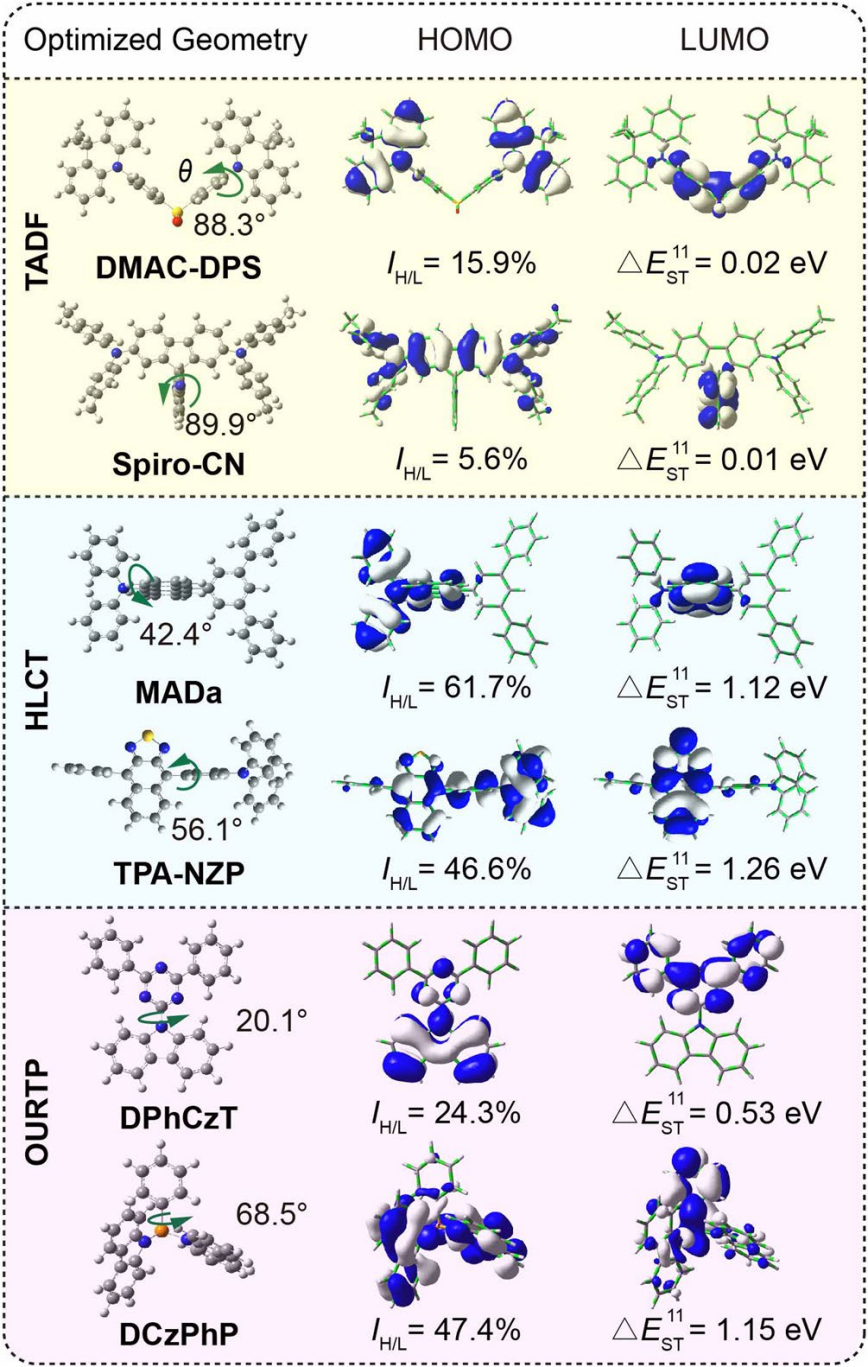
Optimized molecular geometry, frontier orbital electronic density distribution (isovalue = 0.02), *I*_H/L_, and Δ*E*_ST_^11^ of the selected TADF, HLCT and OURTP molecules.

At the ground states (Figs 3 and S1), the donor (D) moieties of dihydroacridine and diphenyl amine in respective TADF molecules of **DMAC-DPS** and **Spiro-CN** are almost orthogonally connected to acceptor (A) moieties with large twisting angles (88.3° and 89.9° respectively), which is important to support the strong charge transfer (CT) feature with small HOMO-LUMO overlap extent (*I*_H/L_) and single-triplet splitting between S_1_ and T_1_ (Δ*E*_ST_^11^) for efficient ISC and RISC processes^31^. In the HLCT molecules of **MADa** and **TPA-NZP**, the twisting angles are decreased to 42.4° and 56.1° with large *I*_H/L_ (61.7% and 46.6%) to harmonize the hybridization of locally excited (LE) and CT states for efficient fluorescence with large transition moment and weakly bound exciton with facile exciton transformation at high-lying excited states, respectively^32^. Whereas, the twisting angles between the D (carbazole) and A (triazine and phenyl phosphine) moiety planes of OURTP molecules can be either large (68.5° in **DCzPhP**) or small (20.1° in **DPhCzT**) with varied *I*_H/L_ of 47.4% and 24.3%. Therefore, although the separated HOMO and LUMO distribution on donor and acceptor moieties with small *I*_H/L_ is important for TADF molecules to achieve small Δ*E*_ST_^11^ with facilitated ISC and RISC processes, efficient singlet/triplet exciton transformation can also occur as found experimentally in HLCT and OURTP molecules with large Δ*E*_ST_^11^ and *I*_H/L_ up to 1.26 eV and 61.7%, respectively.

According to the energy gap law, the initial and final states for the exciton transformation should have similar energy levels and the energy difference must be lower than 0.37 eV^33^; below this energy gap, the transformation can be compensated or dissipated through vibrations of the molecules at room temperature in a thermal equilibrium. For TADF molecules, exciton transformation happens between S_1_ and T_1_^34^, while for HLCT and OURTP molecules, the transformation is from the high-lying T_n_ to S_m_ (n > 1 and m ≥ 1) and from S_1_ to T_n_ (n > 1), respectively^17, 35^. Principally, all of these transformations require a small Δ*E*_ST_. However, to support the high-lying excited state-related transformation as in HLCT and OURTP molecules, the separated HOMO-LUMO distribution strategy in achieving small Δ*E*_ST_ fails. Therefore, it is difficult for frontier molecular orbital analysis to give an overall and convincible description of the exciton transformation processes in HLCT and OURTP molecules.

### Transition Configuration Description

**Figure 4:**
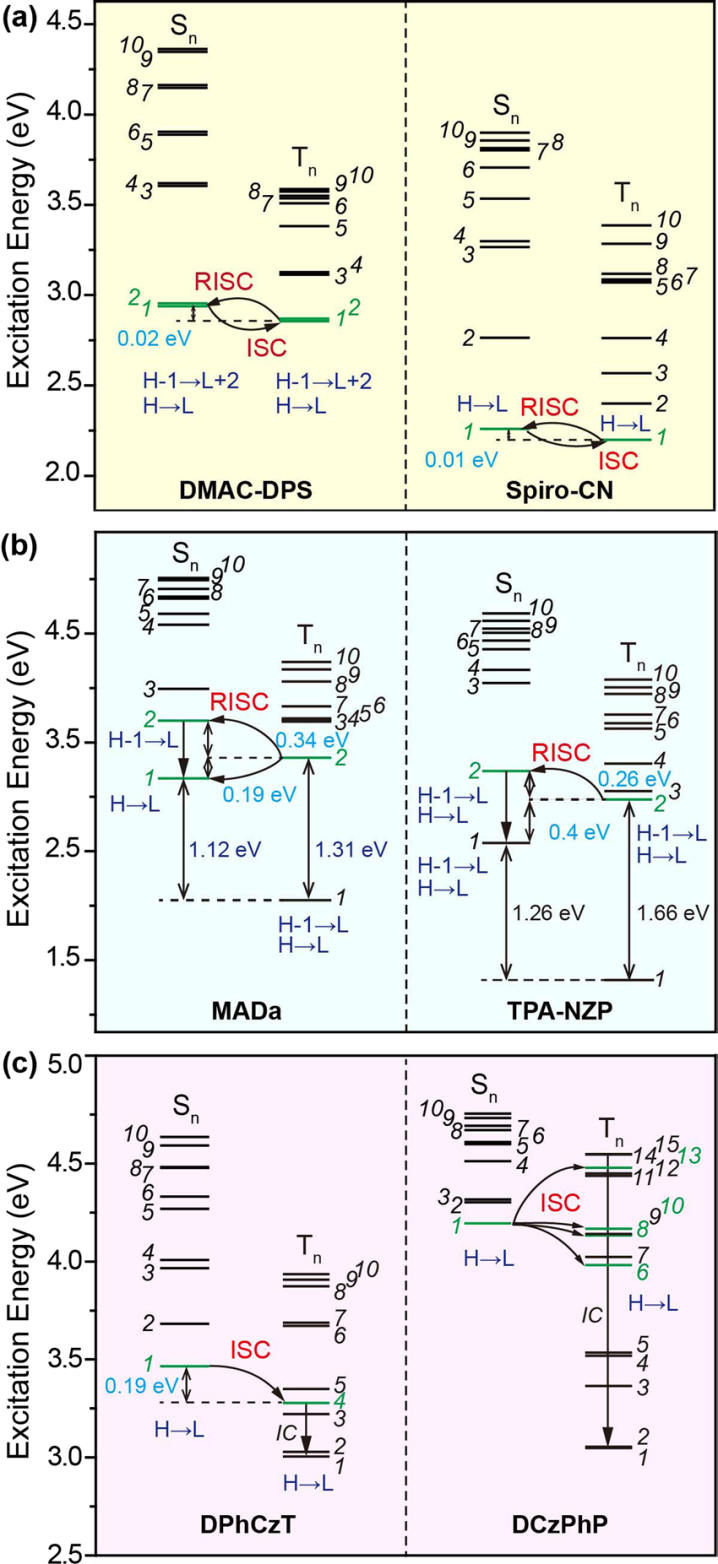
Energy diagrams and transition configurations of singlet (S_n_) and triplet (T_n_) excited states of (**a**) TADF, (**b**) HLCT and (**c**) OURTP molecules. Feasible excited states for exciton transformation are highlighted in green.

We then performed transition configuration investigation of the excited states *via* TD-DFT calculations (Supplementary Tables S2–S7). From Table 1 and Fig. 4, the S_0_ → T_1_ transition configurations are very similar to that of S_0_ → S_1_ in TADF molecules of **DMAC-DPS** and **Spiro-CN**, containing both high HOMO (H) → LUMO (L) components; there are also apparent component overlap with the same transition configurations between S_0_ → S_n_ (n = 1 and 2) and S_0_ → T_1_ in HLCT molecules of **MADa** and **TPA-NZP**; in OURTP molecules (**DPhCzT** and **DCzPhP**), still the same transition configurations can be observed between S_0_ → S_1_ and S_0_ → T_n_ (n = 4, 6, 8, 10, or 13) within the critical energy gap of 0.37 eV. Considering the El-Sayed rule that predicts accelerated ISC by vibronic interactions between (*π*, π*) and (*n*, π*) states^19^, the same transition configuration component of the two excited states indicates their overlapped excitation features, which should be crucial for the enhanced ISC through these allowed transformation channels; and it can be expected that the more overlapped the transition configurations are, the more facile the exciton transformation will be through this channel^36, 37^.

**Table 1:** Excitation energy (*E* in eV) and transition configuration of S_0_ → S_n_ and S_0_ → T_n_ for exciton transformation^a^.

Compound	S_n_			T_n_		
	*n*-th	*E* (eV)	Transition configuration (%)	*n*-th	*E* (eV)	Transition configuration (%)
**DMAC-DPS**	S_1_	2.93	H-1 → L+2 (6.4%), H → L (91.1%)	T_1_	2.91	H-1 → L+2 (6.8%), H → L (90.4%)
	S_2_	2.93	H-1 → L (91.05%), H → L+2 (6.46%)	T_2_	2.91	H-1 → L (90.32%), H → L+2 (6.85%)
**Spiro-CN**	S_1_	2.22	H → L (99.7%)	T_1_	2.21	H → L (98.8%)
**MADa**	S_1_	3.17	H → L (96.8%)	T_2_	3.36	H-1 → L (64.5%), H → L (27.6%)
	S_2_	3.70	H-1 → L (96.1%)			
**TPA-NZP**	S_2_	3.24	H-1 → L (74.9%), H → L (19.6%)	T_2_	2.98	H-1 → L (34.4%), H → L (42.6%)
**DPhCzT**	S_1_	3.47	H → L (98.6%)	T_4_	3.28	H → L (93.9%)
**DCzPhP**	S_1_	4.19	H → L (96.5%)	T_6_	3.98	H → L (27.4%)
				T_8_	4.13	H → L (3.1%)
				T_10_	4.16	H → L (29.5%)
				T_13_	4.47	H → L (2.7%)

^a^H, H-1, L, and L + 2 represent HOMO, HOMO_−1_, LUMO, and LUMO_+2_, respectively.

Specifically, the transition configurations of S_1_ and T_1_ are very similar in TADF molecules; for instance, **DMAC-DPS** has the same transition configurations of 6.4% and 6.8% in H-1 → L + 2 and of 91.1% and 90.4% in H → L for S_1_ and T_1_ respectively, suggesting the very efficient ISC and RISC processes at room temperature as found experimentally^26^. For HLCT molecules, the configuration overlap between T_2_ and S_n_ (n = 1 or 2) is also high, which is over 90% (64.5% + 27.6%) from the sum of the two lower transition configurations in **MADa** and 77.0% (34.4% + 42.6%) in **TPA-NZP** for T_2_ → S_2_; it should be noted that a high energy barrier between T_2_ and T_1_ states is required to hinder the rapid internal relaxation to maintain the stable T_2_ with high exciton population for RISC^11^. In OURTP molecules, overlapped transition configuration between S_1_ and T_n_ was obvious too, reaching 93.9% in **DPhCzT** and 29.5% in **DCzPhP** respectively; the lower configuration overlap in **DCzPhP** may be a reason for its relatively weaker organic afterglow emission^17^.

Taken both the energy gap and transition configuration together, a universal exciton transformation mechanism for TADF, HLCT, and OURTP molecules can be proposed (Fig. 4). Firstly, the molecules should contain heteroatoms to facilitate the exciton transformation according to the El-Sayed rule. Secondly, the singlet and triplet excited states should be close in energy with Δ*E*_ST_ lower than 0.37 eV, otherwise the transformation cannot be fueled by the environmental thermal energy at room temperature or facilely disperse the excess energy *via* molecular vibrations. Thirdly, the two excited states should contain the same components of transition configurations to establish the transformation channels in bridging the spin-forbidden transitions between two electronic states with different spin multiplicities. Without the matched energy gap, the excitons are weak in transformation due to the lack of enough energies achievable from thermal vibrations or suitable ways in dissipating excess energies. Without the matched transition configuration, the excitons are locked by the forbidden spin state change for transformation. Only when both the matched energy gap and transition configuration with high heteroatom participation are satisfied, facile exciton transformation *via* ISC and RISC processes is possible in purely organic optoelectronic molecules.

### Natural Transition Orbital (NTO) Analysis

**Figure 5:**
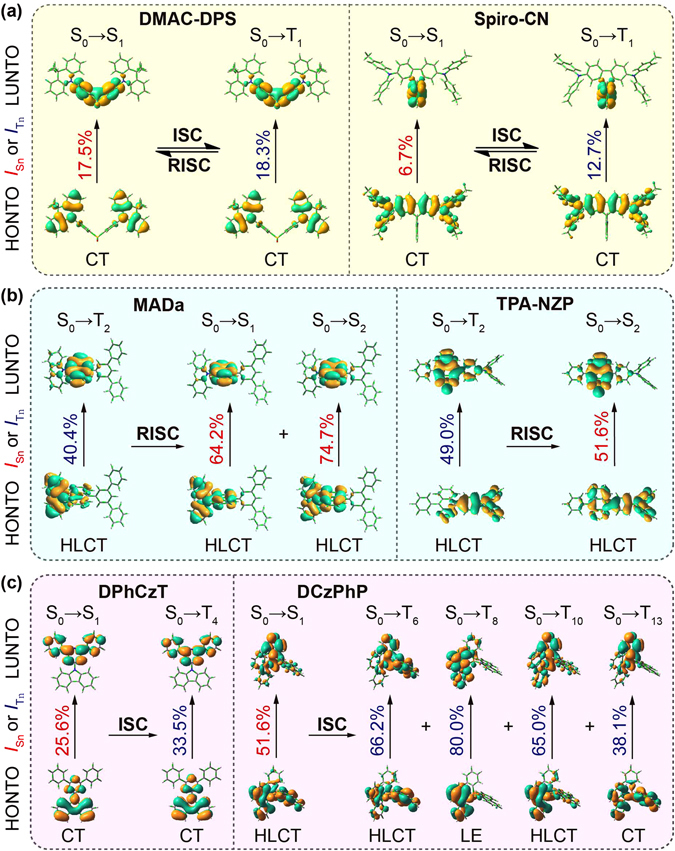
HONTO and LUNTO distributions and overlap extents of the singlet (S_n_) and triplet (T_m_) states for exciton transformation in (**a**) TADF, (**b**) HLCT and (**c**) OURTP molecules.

To give a whole picture of exciton transformation, natural transition orbital (NTO) analysis based on the singular value decomposition of 1-particle transition density matrix was performed. The resulting compact frontier orbitals can represent any one electron property associated with the electronic transition. In principle, the highest occupied natural transition orbital (HONTO) to the lowest unoccupied natural transition orbital (LUNTO) excitation amplitude is always the most significant for any particular excited state, due to its dominating role in determining the one electronic transition for the generation of the corresponding excited state from the ground state (S_0_)^25^. HONTOs and LUNTOs of all the studied six molecules at the lowest 10~15 singlet and triplet excited states were investigated (Figs S2–S7). From the singlet/triplet excited state pairs that are possible in energy for exciton transformation according to the energy gap law (|Δ*E*_ST_| < 0.37 eV) (Fig. 5a), very similar HONTO and LUNTO distributions at both singlet and triplet excited states were observed in TADF molecules, where donor moiety dominates HONTO and acceptor moiety determines LUNTO for very small overlap between HONTO and LUNTO. The almost identical HONTO and LUNTO distributions for S_0_ → S_1_ and S_0_ → T_1_ may indicate facile exciton transformation channel for efficient ISC and RISC processes between S_1_ and T_1_, which are coincident with the experimental observations of the TADF molecules^38^. For HLCT molecules, LUNTO distributions are mainly located at the acceptor moieties, while HONTOs of singlet excited states have larger contributions from the weak acceptor moieties than that of triplet excited states, leading to the partially overlapped HONTO and LUNTO distributions for HLCT at both singlet and triplet excited states (Fig. 5b). As to OURTP molecules, HONTO and LUNTO distributions can be either separated (**DPhCzT**) or overlapped (**DCzPhP**), but they should be similar enough at the two singlet and triplet excited states, *i.e*. the HONTO (and LUNTO) at S_1_ is well overlapped with HONTO (and LUNTO) at T_1_, to support the exciton transformation channels for ISC (Fig. 5c).

Furthermore, in order to give a quantitative evaluation of HONTO-LUNTO overlap, the overlap extents at both S_n_ (*I*_S_) and T_n_ (*I*_T_) states were calculated using Multiwfn^36, 39^. The separated HONTO and LUNTO distributions lead to small *I*_S_ and *I*_T_, while large *I*_S_ and *I*_T_ represent heavily overlapped HONTO and LUNTO. Interestingly, small *I*_S_ and *I*_T_ were observed in TADF molecules; larger I_S_ and *I*_T_ were found in HLCT molecules; but OURTP molecules show both small and large *I*_S_ and *I*_T_. The key point is that *I*_S_ and *I*_T_ should be close in value for all the three kinds of materials, suggesting that the close values of *I*_S_ and *I*_T_ are a direct result of the similar HONTO and LUNTO distributions at singlet and triplet excited states. Based on these values, CT (0~40%), HLCT (40~75%), and LE (75~100%) characters of both singlet and triplet excited states can be quantitatively classified. In this sense, both S_1_ and T_1_ need to be in CT with low *I*_S_ and *I*_T_ for TADF molecules; HLCT character of T_n_ (n > 1) with moderate *I*_T_ is essential for HLCT molecules to transform to singlet excited states with both CT and LE characters, since HLCT contains both LE and CT components^29^. For CT characterized OURTP molecule of **DPhCzT**, both S_1_ and T_4_ should be CT with low *I*_S_ and *I*_T_, while for HLCT type OURTP molecule of **DCzPhP**, the HLCT characterized S_1_ offers many transformation channels to all the CT, HLCT, and LE characterized triplet excited states with close *I*_T_ values to *I*_S_.

### Excited State Similarity (s)

Since the HONTO and LUNTO describe the transition features of an excited state in a whole, similar HONTO and LUNTO between the singlet and triplet excited states means the similar excitation feature, which would essentially provide effective channels for the exciton transformation between them. Therefore, we developed a new formula in equation (3) to evaluate the excited state similarity (*s*):

3 $${s}_{{\rm{H}}/L}=1-\frac{\sum _{i}|{a}_{i}-{b}_{i}|}{2}$$

where Σ_i_ *a*_i_ = 1 and Σ_i_ *b*_i_ = 1. The index *i* is the number of atoms in the molecule; *a*_i_ and *b*_i_ are the contribution percentages of different atoms in the frontier NTO of the corresponding singlet and triplet excited states, respectively^40^. This orbital composition analysis was done using Multiwfn. |*a*_i _− *b*_i_| denotes the contribution percentage difference of an atom (*i*) in HONTO (or LUNTO) between the singlet and triplet excited states.

The *s*_H_ and *s*_L_, which describe quantitatively the orbital similarity in HONTO (*s*_H_) and LUNTO (*s*_L_), offer new parameters in evaluating the exciton transformation of organic molecules. Large values of *s*_H_ and *s*_L_ indicate high similarity of HONTO and LUNTO between the singlet and triplet excited states. For TADF, HLCT, and OURTP molecules capable of efficient exciton transformation as found experimentally, both high *s*_H_ and *s*_L_ should be observed between singlet and triplet excited state pairs with energy gap below 0.37 eV^33^; and the larger values of *s*_H_ and *s*_L_, the more facile the transformation channels will be. With this basic consideration, possible exciton transformation channels in all the studied molecules can be picked out by scanning both the energy gap and transition similarity (*s*_H_ and *s*_L_). Since higher *s* means more facile intersystem crossing channel, it should be reasonable that the values of *s*_H_ and *s*_L_ must be both larger than 0.50 for efficient exciton transformation. To our delight, the predicted possible exciton transformation channels are well in line with that obtained through above transition configuration analysis *via* TD-DFT calculations, except for S_1_ → T_8_ of **DCzPhP** and T_2_ → S_2_ of **MADa** (Fig. 5 and Supplementary Table S8). The transformation *via* S_1_ → T_8_ in **DCzPhP** and T_2_ → S_2_ in **MADa** is suggested to be possible for ISC by TD-DFT analysis, but their *s*_L_ or *s*_H_ is too low (*s*_L_ = 0.175 and *s*_H_ = 0.468) to support the efficient exciton transformation. These two differences are understandable, because the S_1_ → T_8_ transformation channel in **DCzPhP** must be very weak with H → L ratio of only 3.1%, while in the case of T_2_ → S_2_ of **MADa**, its energy gap is high up to 0.34 eV, suggesting the high energy barrier in exciton transformation *via* this channel.

**Figure 6:**
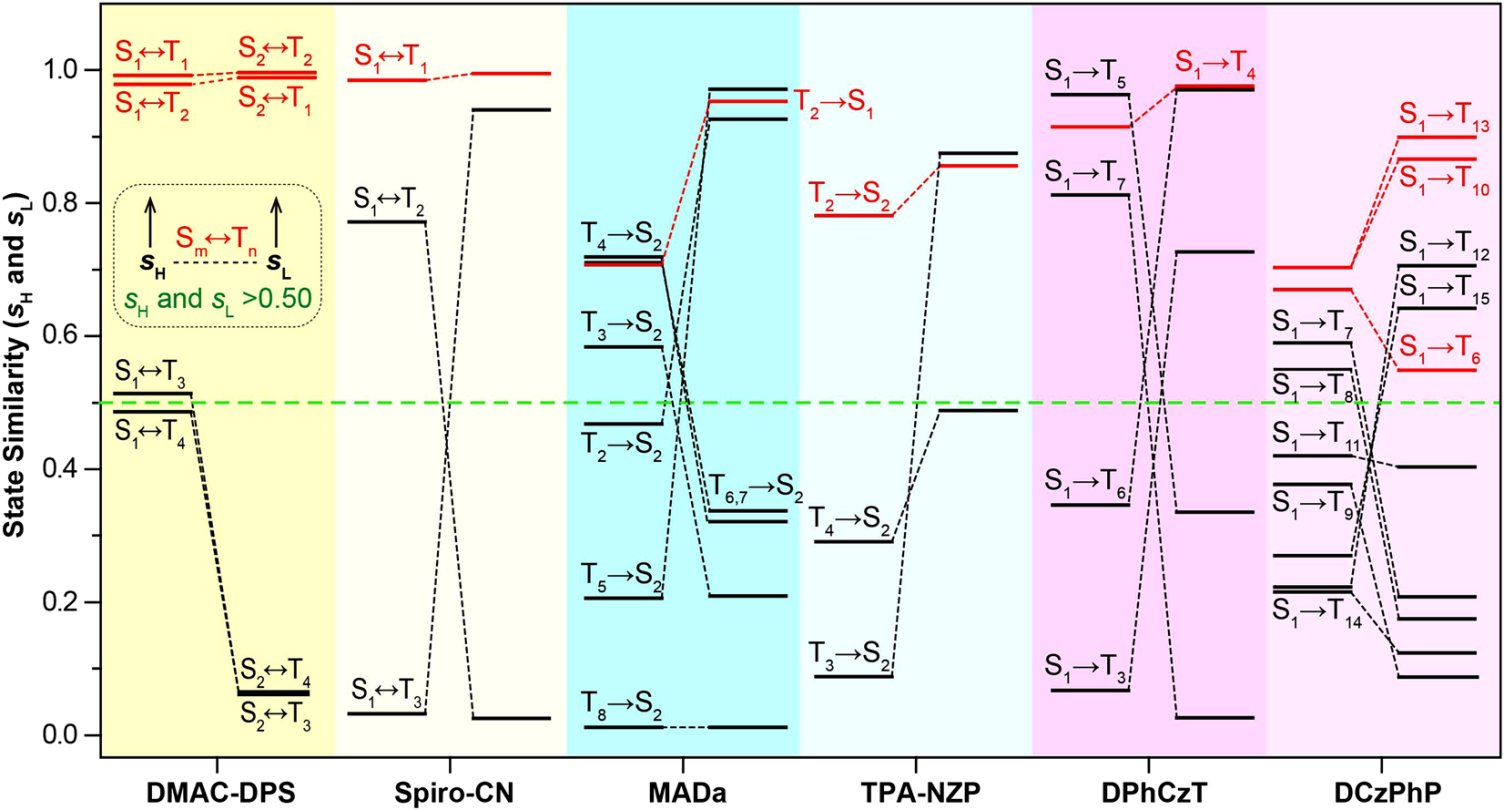
Similarity of HONTO (*s*_H_, left) and LUNTO (*s*_L_, right) between S_m_ and T_n_ in TADF (S_m_ ↔ T_n_), HLCT (T_n_ → S_m_), and OURTP (S_1_ → T_n_) molecules. The matched excited states for efficient exciton transformation that have the small energy gap (|Δ*E*_ST_| < 0.37 eV) and high state similarity (*s*_H_ and *s*_L_ > 0.50) are highlighted in red.

It is clear that both ISC and RISC happen between S_1_ and T_1_ in TADF molecules, but in HLCT and OURTP molecules, the transformation channels cannot be easily identified, although the ISC and RISC do proceed efficiently from experimental evidences. With our new-proposed parameters, it is possible to describe the excited state similarity quantitatively and more interestingly, to figure out the transformation channels and their feasibility. From Fig. 6, highly efficient channels for exciton transformation are all observed, exhibiting high transition similarity (*s*_H_ and *s*_L_ > 0.50) to support both ISC and RISC processes in these molecules. Four channels (S_1_ ↔ T_1_, S_1_ ↔ T_2_, S_2_ ↔ T_1_, and S_2_ ↔ T_2_) with very high *s*_H_ and *s*_L_ (>0.95) are found to be available for the facile exciton transformation in the TADF molecule of **DMAC-DPS**; this should be an important reason for its high TADF performance in device applications^26^. The highest *s*_H_ and *s*_L_ found in TADF molecules suggest the existence of the facile transition-allowed channels for both ISC and RISC, which is in line with the experimental results (EQE≈30%)^10^. In HLCT molecules, *s*_H_ and *s*_L_ are slightly reduced, and they can be either high or low in OURTP molecules, suggesting relatively lower exciton transformation efficiency in these molecular systems; this understanding well explains the lower OLED performance (EQE≈8%) of HLCT molecules^41^ and the weak ultralong-lived phosphorescence of OURTP molecules observed experimentally^42^.

To test the validity of our method in predicting the exciton transformation channels and efficiencies, the SOC values of **DPhCzT** of various transformation channels from S_1_ to T_n_ were calculated using Dalton package (B3LYP/cc-pVTZ) based on its optimized S_1_ state structure. From Supplementary Table S9, the S_1_ → T_3_ and S_1_ → T_4_ channels have relatively high SOC values, which is matched with our predictions of S_1_ → T_4_. Although our qualitative method cannot predict the high SOC of S_1_ → T_3_ suggested by the Dalton calculation, our method succeeds in figuring out efficient SOC channels for exciton transformation. To further identify the proportion of (*n, π* ^*^) configuration (*α*_n_%) of the excited states, Mulliken population analysis (MPA) was performed on the optimized excited state structures with the aid of Multiwfn package^16^. The S_1_ of **DPhCzT** was found to have a high component of ^1^(*n, π* ^*^) with *α*_n_% of 14.3%, while the T_4_ is mainly ^3^(π, π*) with *α*_n_% of 0.0%; Such a significant *α*_n_% change (Δ*α*_n_ = |*α*_n,S1_ − *α*_n,T4_ | = 14.3%) suggests the allowed ^1^(*n*, *π* ^*^) → ^3^(*n, π* ^*^) transition from S_1_ to T_4_ according to El-Sayed rule, which is also coincident with the results of our TD-DFT calculation method based on the ground state geometry (S_0_).

Furthermore, a control calculation was performed based on a pair of molecules with and without exciton transformation properties. According to the literature report^43^, Compound **1** has very slow intersystem crossing rate (*k*_ISC_), while the exciton transformation in Compound **2** is very efficient (Supplementary Table S10), although they have quite similar molecular structure (Fig. S8). According to our method, only one weak S_1_ → T_3_ transformation channel (2.92%) can be observed in compound **1**, while Compound **2** has three available channels and that of S_1_ → T_1_ and S_1_ → T_5_ transformations are highly efficient with high transition configuration component overlap (67.45% and 21.53%, respectively) (Supplementary Tables S11 and S12). These theoretical results predict well the experimentally observed very small *k*_ISC_ in Compound **1** and high *k*_ISC_ in Compound **2**, suggesting clearly that our method is reliable in predicting the exciton transformation channels for intersystem crossing.

Looking further ahead, these new insights into exciton transformation principles could suggest a basic molecular design guidance for the TADF, HLCT and OURTP materials. Relatively strong donor and acceptor moieties should be used in constructing high-performance TADF molecules for well-separated HONTO and LUNTO at both S_1_ and T_1_ states with small Δ*E*_ST_^11^, *I*_S_ and *I*_T_; one weak donor or acceptor moiety is important for HLCT molecules to form HLCT state that contains both CT and LE components with large Δ*E*_ST_^11^, large T_2_ to T_1_ energy gap, and moderate *I*_S_ and *I*_T_ values; either strong or weak donor and acceptor moieties can be used to design CT- or HLCT- type OURTP molecules with small or large *I*_S_ and *I*_T_, respectively^42^. The use of donor and acceptor moieties to construct a CT state with low exciton binding energy is essential for exciton transformation, because the weakly bound exciton at CT states has similar energies at singlet and triplet state for small Δ*E*_ST_, but excitons at LE state with high binding energy will significantly hinder the spin flip process for exciton transformation. However, the large transition moment from LE state is helpful for a high-efficiency fluorescence radiative decay, and may be a reason for large energy gaps between the triplet excited states, which are very important for luminescent materials and highly populated high-lying excited states for “hot exciton” transformation^32^. HLCT, contains both CT and LE components, seems to be recommendable in constructing high-performance optoelectronic materials, intending to use its CT component to facilitate the exciton transformation and LE component for high luminescence; but special attention should be paid to let them work exactly as expected, avoiding the disadvantages from both sides of the two components^44^.

## Discussion

We have presented a systematic theoretical investigation to figure out crucial factors in influencing the exciton transformation with the aid of a facile and straightforward computational method developed to study ISC or RISC processes in recently emerged TADF, HLCT, and OURTP molecules with efficient exciton transformation feature. Besides the well-recognized importance of energy gap and heteroatom participation for exciton transformation, the significance of excited state transition configurations in supporting feasible transformation channels was identified using quantitative parameters of *I*_S_, *I*_T_, *s*_H_ and *s*_L_ through TD-DFT calculations and NTO analysis at the optimized ground state structures. Both large *s*_H_ and *s*_L_ are required to support facile channels for exciton transformation, while values of *I*_S_ and *I*_T_ are useful in quantitatively classifying CT, HLCT, and LE states to provide a general guidance for the molecular design of the materials with desired exciton transformation properties. Both energy gap and vertical transition configuration of the excited states need to be considered in supporting the exciton transformation between them. Our method, which is based on the ground state molecular geometry, is coincident with El-Sayed rule and could be considered as an alternative way in evaluating the excited states and their ISC processes for exciton transformation. These findings, as verified in three kinds of material systems, should shed important light on the fundamental singlet/triplet exciton transformation mechanism of organic molecules, stimulating further the research of purely organic materials capable of facile exciton transformation.

## Methods

DFT and TD-DFT calculations were performed to investigate the singlet/triplet exciton transformation using Gaussian 09 package. The molecular geometries in the ground state (S_0_) were optimized *via* spin-restricted formalism at the B3LYP/6-31 G(d) level of theory. Vibrational frequency calculations were carried out to test that the optimized structures are truely corresponding to the minima on the potential energy surfaces. The excited singlet (S_n_) and triplet (T_n_) states were investigated by the time-dependent DFT (TD-DFT) formalisms of B3LYP (20% HF), PBE0 (25% HF), BMK (42% HF), M06–2X (56% HF), and M06-HF (100% HF) functionals with 6–31 G(d) basis set were performed based on the optimized ground-state geometries to investigate the vertical excited energies. The vertical excitation energies of the studied molecules were also evaluated using the range separated hybrid exchange functional of ωB97XD, which consists in a mix of short range density functional exchange with long range Hartree-Fock exchange (22% HF at short range and 100% at long range). To obtain a precise picture of the excited states, natural transition orbitals (NTOs) analysis were further performed to give a compact orbital representation for the electronic transition density matrix. The SOC values between S_1_ and T_n_ were calculated using Dalton package (B3LYP/cc-pVTZ) based on the optimized S_1_ state structure. The proportion of ^1^(n, π*) configuration (*α*_n_%) of the excited state was calculated by using Mulliken population analysis (MPA) to identify the *n* orbital components of the frontier orbitals at the optimized excited state geometries with the aid of Multiwfn package^16^.

## Electronic supplementary material

Supplementary information
