# Supplementary material for: Promoting Singlet/triplet Exciton Transformation in Organic Optoelectronic Molecules: Role of Excited State Transition Configuration
Source: Sci Rep. 2017 Jul 24;7:6225. doi: 10.1038/s41598-017-05339-4 (PMC5524908; doi:10.1038/s41598-017-05339-4)
Supplement: Supplementary file 1 — Supplementary information [file 41598_2017_5339_MOESM1_ESM.pdf]

Supplementary Information for

Promoting Singlet/triplet Exciton Transformation of Organic Optoelectronic Molecules: Role of Excited State Transition Configuration

Runfeng Chen[1*], Yuting Tang[1], Yifang Wan[1], Ting Chen[1], Chao Zheng[1*], Yuanyuan Qi[1], Yuanfang Cheng[1] & Wei Huang[1,2*]

[1]*Key Laboratory for Organic Electronics and Information Displays & Institute of Advanced Materials, Jiangsu National Synergetic Innovation Center for Advanced Materials, Nanjing University of Posts & Telecommunications, 9 Wenyuan Road, Nanjing 210023, China*

[2]*Key Laboratory of Flexible Electronics & Institute of Advanced Materials, Jiangsu National Synergetic Innovation Center for Advanced Materials, Nanjing Tech University, 30 South Puzhu Road, Nanjing 211816, China*

Contents

1. **Computational Details**
2. **The singlet-triplet splitting (ΔE_{ST})**
3. **The calculation of overlap integral**
4. **Natural transition orbital (NTO) analysis**
5. **Excited state similarity (s)**
6. **Supplementary figure**
7. **Supplementary tables**

References

1. Computational Details

The density functional theory (DFT)[1] and time-dependent density functional theory (TD-DFT)[2] calculations were performed to investigate the singlet/triplet exciton transformation using Gaussian 09 package.[3] The Becker's three-parameter exchange functional along with the Lee Yang Parr's correlation functional (B3LYP) that can well predict the geometric structures of organic molecules,[4] was adopted to optimize the ground state (S_0) geometries of all molecules in conjunction with the 6-31G(d) basis set. The optimized structures were further characterized by harmonic vibrational frequency analysis to confirm that real local minima without any imaginary frequency was reached at the same computational level. TD-DFT calculations by the B3LYP (20% HF),[5] PBE0 (25% HF),[6] BMK (42% HF),[7] M06-2X (56% HF),[8] and M06-HF (100% HF)[9] functionals with 6-31G(d) basis set were performed based on the optimized ground-state geometries to investigate the vertical excited energies and the singlet-triplet energy splitting (ΔE_{ST}) (**Table S1**). The vertical excitation energies of the studied molecules were also evaluated using the range separated hybrid exchange functional of ωB97XD,[10] which consists in a mix of short range density functional exchange with long range Hartree-Fock exchange (22% HF at short range and 100% at long range).

To get further insights into the nature of the excited states, NTOs analysis was performed based on TD-DFT results to offer a compact orbital representation for the electronic transition density matrix.[11] In addition, using the overlap integral function embedded in Multiwfn,[12] the overlap integrals of $I_{H/L}$, I_S and I_T between the highest occupied molecular orbital (HOMO) and the lowest unoccupied molecular orbital (LUMO), the highest occupied NTO (HONTO) and the lowest unoccupied NTO (LUNTO) at the singlet excited states (S_n) and triplet excited states (T_n) of investigated molecules can be calculated respectively. Excited state similarity was evaluated through the contribution percentage difference between the singlet and triplet excited states in HONTO (s_H) and LUNTO (s_L).

Spin-orbit coupling (SOC) matrix elements between the singlet and triplet excited states are calculated with quadratic response function methods using the

Dalton program.[13] The SOCs of **DPhCzT** were performed at the optimized geometry of the first singlet excited state (S_1) using B3LYP functional and cc-pVTZ basis set.

To identify proportion of (n, π^*) configuration (α_n%) of the excited states, Mulliken population analysis (MPA) was performed to calculate the n orbital components with the aid of Multiwfn package.[14] As exemplified in **DPhCzT**, the lone-pair electrons of N atom in carbazole are localized on p_z, while that of three N atoms in triazine are on p_x and p_y, since all the N atoms of **DPhCzT** are sp^2 hybridized. The single-center $p_{x,y} \leftrightarrow p_z$ transition can promote the SOC for efficient exciton transformation. Therefore, the n orbital components were calculated as the sum of p_z of N atom in carbazole and p_x and p_y of N atoms in triazine. Based on the optimized geometries of S_1 and T_4 excited states at PBE0/6-31G(d) level, the S_1 of **DPhCzT** was found to have a high component of $^1(n, \pi^*)$ with n orbital proportion (α_n%) of 14.3%, while the T_4 is mainly $^3(\pi, \pi^*)$ with α_n% of 0.0%.

2. The singlet-triplet splitting (ΔE_{ST})

Theoretically, the singlet-triplet energy splitting ΔE_{ST} is controlled by the electron-exchange energy, which is the twice of the exchange integral (J)[15] in value as described in equation (S1), resulting from the repulsion interaction of the two unpaired electrons (with electric charge of e) on the HOMO (φ_H) and LUMO (φ_L) orbitals as shown in equation (S2).

$$\Delta E_{\mathrm{ST}} = E_{\mathrm{S1}} - E_{\mathrm{T1}} = 2J \tag{S1}$$

$$J = \iint \varphi_L(1)\varphi_H(1)\left(\frac{e^2}{r_1 - r_2}\right)\varphi_L(2)\varphi_H(2)\, dr_1\, dr_2 \tag{S2}$$

$$\Delta E_{\mathrm{ST}} = \iint \varphi_L(1)\varphi_H(1)\left(\frac{2e^2}{r_1 - r_2}\right)\varphi_L(2)\varphi_H(2)\, dr_1\, dr_2 \tag{S3}$$

From equation (S2), J is determined by spatial separation (r_1-r_2) and overlap integral of φ_H and φ_L, *i.e.*, spatial wave function separation of frontier orbitals.[16] In principle, a small overlap or a large separation between HOMO and LUMO will lead to a small ΔE_{ST} and vice versa.[17]

3. The calculation of overlap integral

Using the overlap integral function embedded in Multiwfn,[12] the overlap between two orbitals (φ_i and φ_j) of a molecule can be calculated as illustrated in equation (S4).

$$I = \int |\varphi_i(r)||\varphi_j(r)| dr \tag{S4}$$

For example, the overlap integral of HOMO (φ_H) and LUMO (φ_L) is

$$I_{H/L} = \int |\varphi_H(r)||\varphi_L(r)| dr \tag{S5}$$

More details about the overlap integral can be found in Multiwfn manual.[18]

4. Natural transition orbital (NTO) analysis

Natural transition orbitals (NTOs) can offer a compact orbital representation for the electronic transition density matrix (T), which is diagonal with a dimension of $N_{occ.} \times N_{virt.}$ (N_{occ}, number of occupied orbitals; N_{virt}, number of virtual orbitals):

$$\left| \left[U^{\dagger} T V \right]_{ij} \right| = \lambda_i \delta_{ij} \tag{S7}$$

where, U and V are square unitary transformation matrices of dimensions $N_{occ.} \times N_{occ.}$ and $N_{virt.} \times N_{virt.}$, respectively; $U^{\dagger}$ denotes the conjugate transpose of matrix U; λ_i represents the singular value of matrix T; δ_{ij} is the Kronecker delta. Notably, all one electron properties associated with the transition can be interpreted in a transparent way as a sum over the occupied natural transition orbitals, each orbital being paired with a single unoccupied orbital and weighted with the appropriate eigenvalue λ_i. Hence, the NTO analysis can provide a compact description of an excited state with fewer orbital pairs than the ones given on the basis of frontier molecular orbitals.[11]

Similarly to equation (S4), the overlap integral of the highest occupied NTO (HONTO) ($\varphi_{H'}$) and the lowest unoccupied NTO (LUNTO) ($\varphi_{L'}$) at S_n or T_n states described by NTO analysis can be calculated in equations (S8) and (S9), respectively.

$$I_S = \int |\varphi_{H'}^{S}(r)||\varphi_{L'}^{S}(r)| dr \tag{S8}$$

$$I_T = \int |\varphi_{H'}^{T}(r)||\varphi_{L'}^{T}(r)| dr \tag{S9}$$

5. Excited state similarity (*s*)

Following the previously developed calculation method of charge transfer amount,[19] excited state similarity in HONTO (s_H) and LUNTO (s_L) between the

singlet and triplet excited states can be calculated according to equation (S10):

$$s_{H/L} = 1 - \frac{\sum_i |a_i - b_i|}{2} \tag{S10}$$

where $\Sigma_i a_i=1$ and $\Sigma_i b_i=1$. The index i is the number of atoms in the molecule; a_i and b_i are the contribution percentages of different atoms in the frontier NTO of the corresponding singlet and triplet excited states, respectively. This orbital composition analysis was done by using Multiwfn.[12] $|a_i-b_i|$ denotes the contribution percentage difference of an atom (i) in the HONTO (or LUNTO) between the singlet and triplet excited states.

6. Supplementary figures

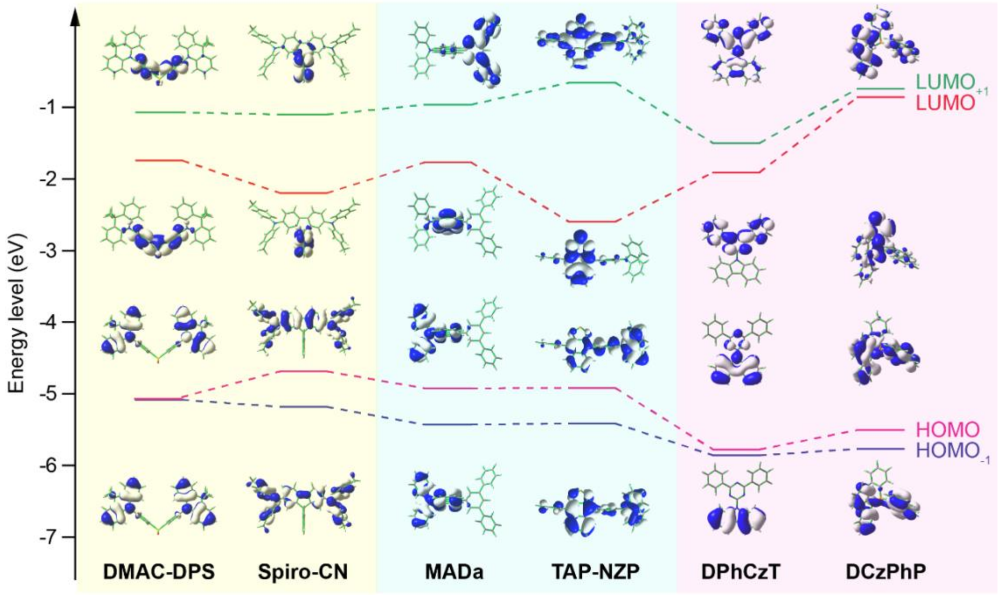

Figure S1. B3LYP/6-31G(d) calculated frontier orbital ($HOMO_{-1}$, HOMO, LUMO and $LUMO_{+1}$) energy levels and spatial plots (isovalue 0.02) of **DMAC-DPS**, **Spiro-CN**, **MADa**, **TAP-NZP**, **DPhCzT** and **DCzPhP**.

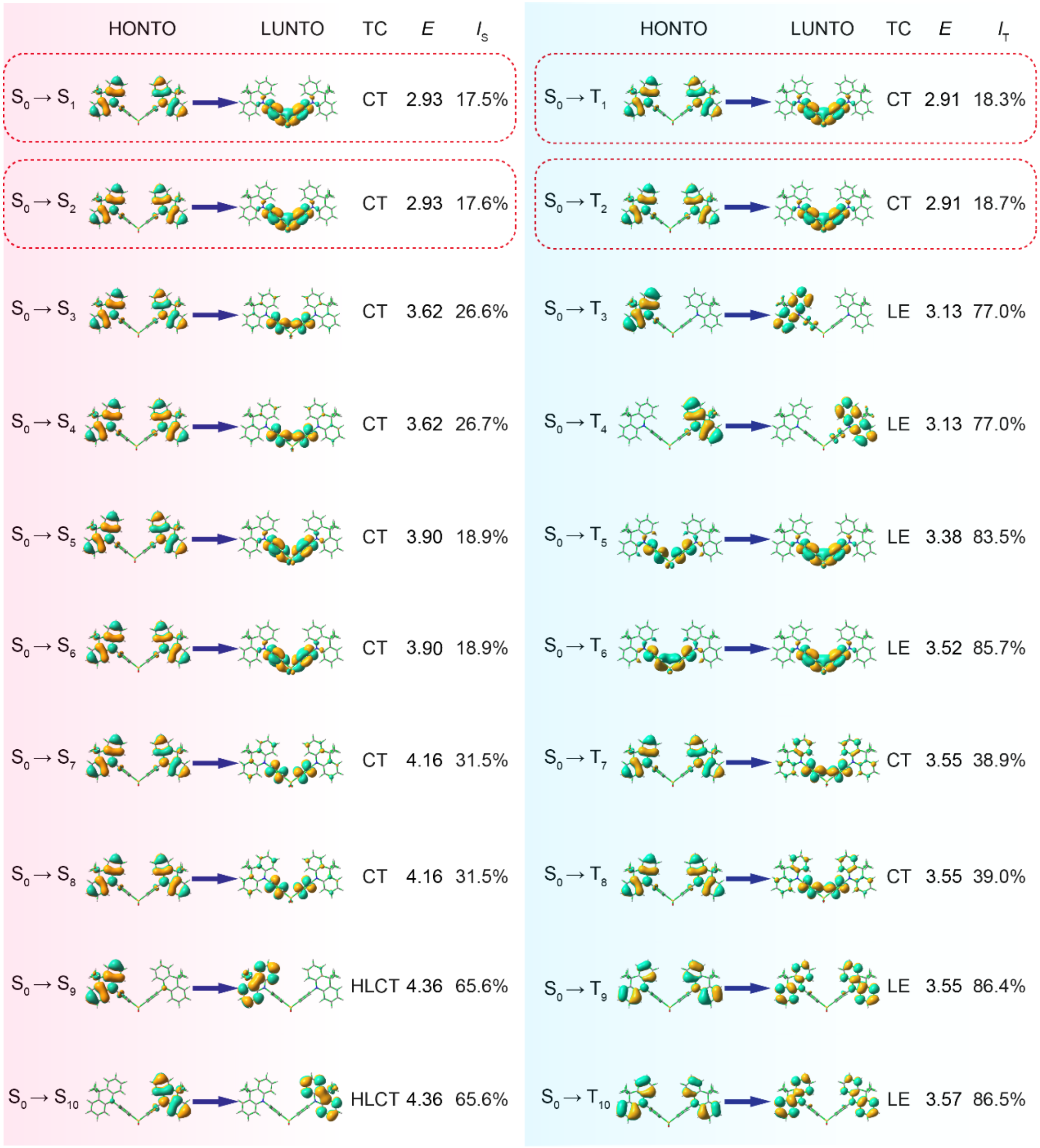

Figure S2. Excitation energy (*E*, in eV), HONTO and LUNTO distribution, HONTO-LUNTO overlap extent, and transition character (TC) of the singlet (left, S_1~S_{10}) and triplet (right, T_1~T_{10}) excited states of **DMAC-DPS**.

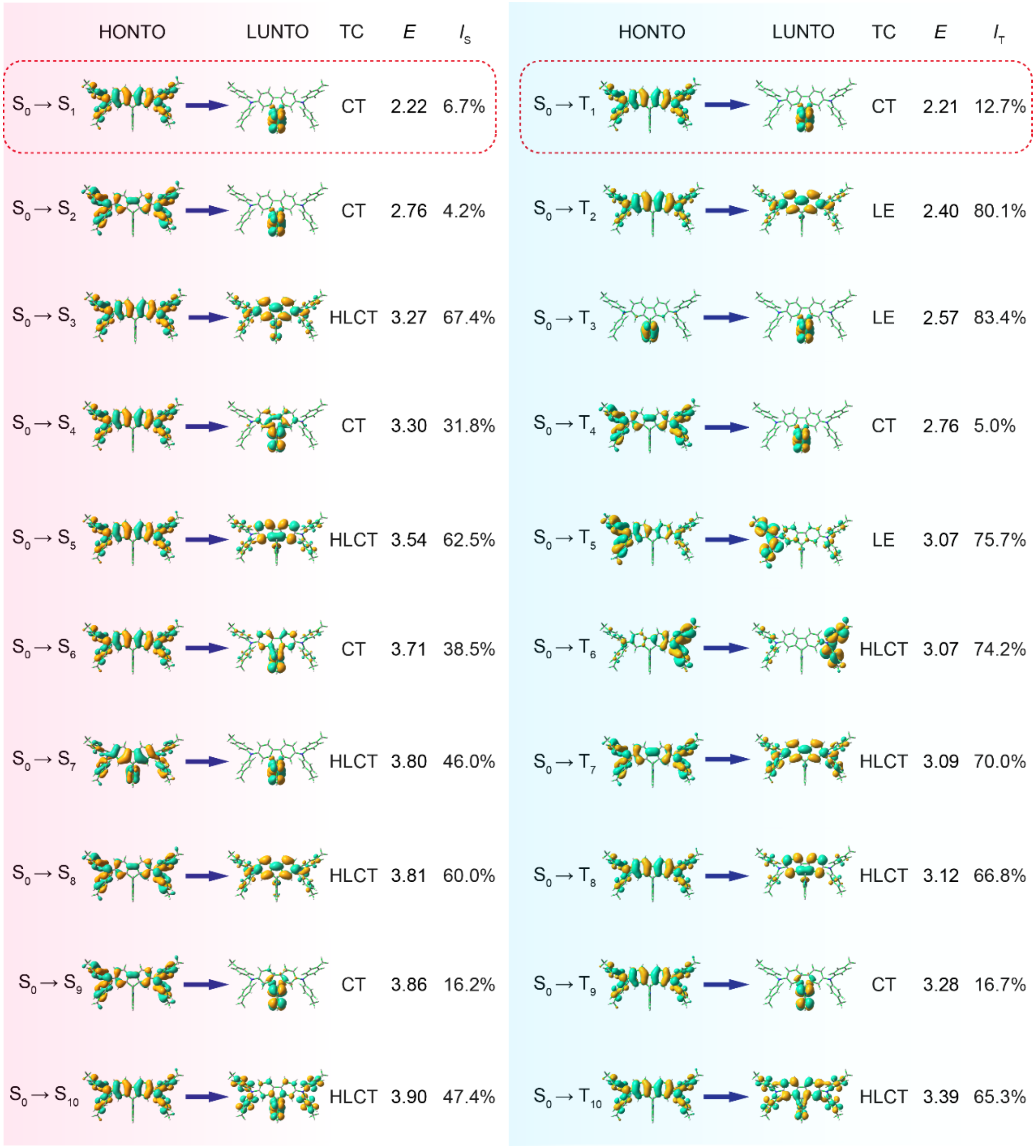

Figure S3. Excitation energy (*E*, in eV), HONTO and LUNTO distribution, HONTO-LUNTO overlap extent, and transition character (TC) of the singlet (left, S_1~S_{10}) and triplet (right, T_1~T_{10}) excited states of **Spiro-CN**.

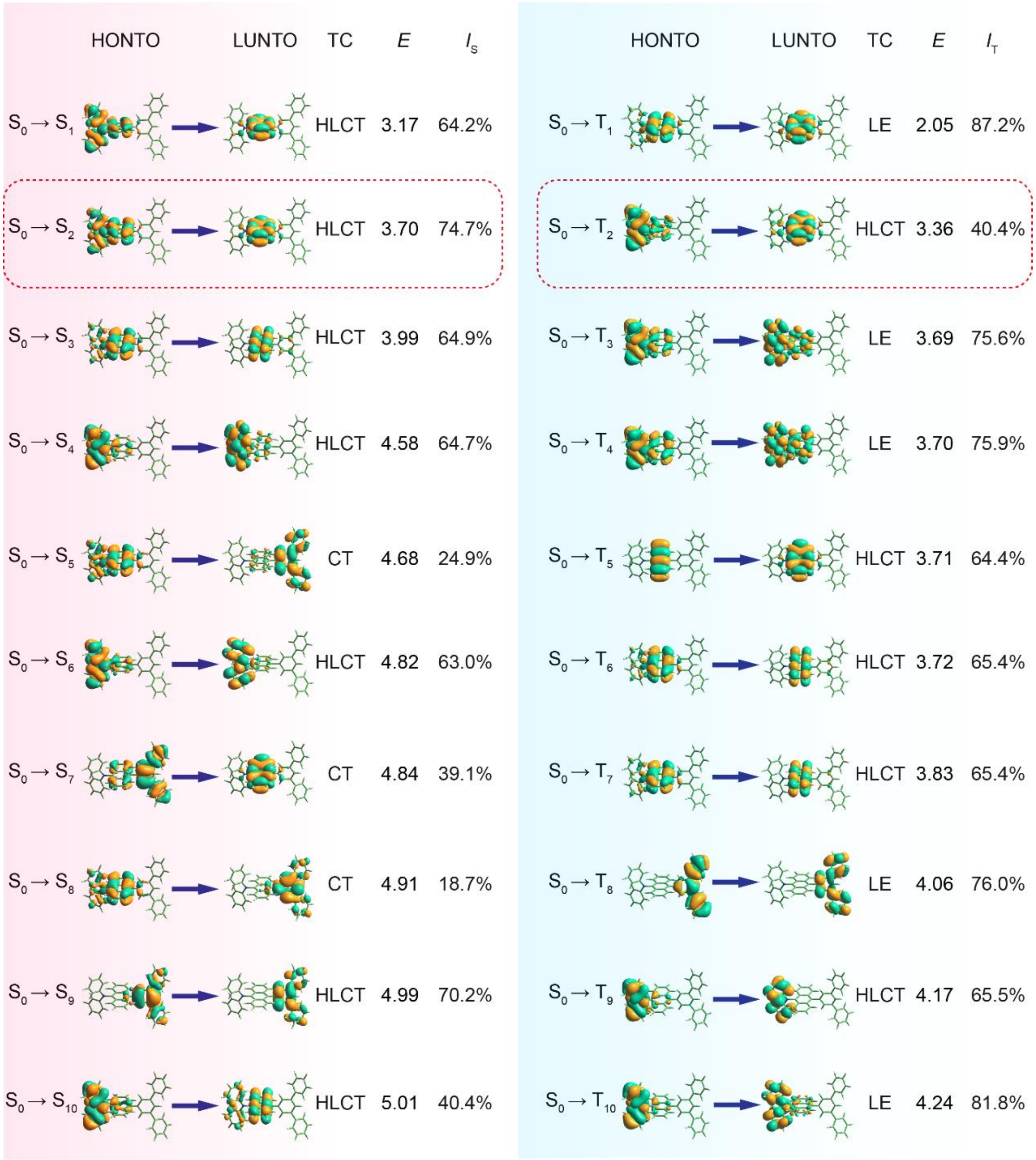

Figure S4. Excitation energy (*E*, in eV), HONTO and LUNTO distribution, HONTO-LUNTO overlap extent, and transition character (TC) of the singlet (left, S_1~S_{10}) and triplet (right, T_1~T_{10}) excited states of **MADa**.

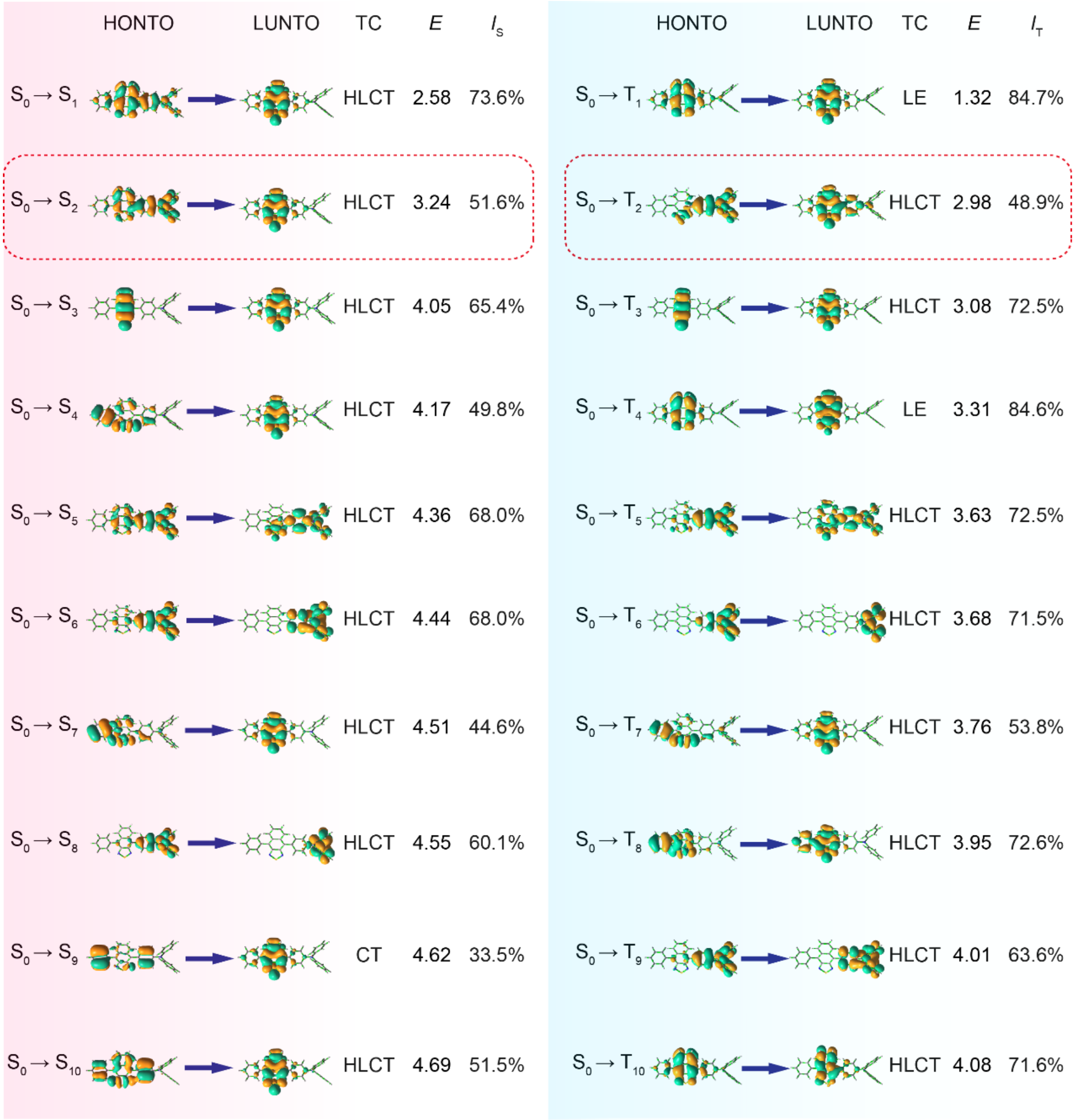

Figure S5. Excitation energy (*E*, in eV), HONTO and LUNTO distribution, HONTO-LUNTO overlap extent, and transition character (TC) of the singlet (left, S_1~S_{10}) and triplet (right, T_1~T_{10}) excited states of **TPA-NZP**.

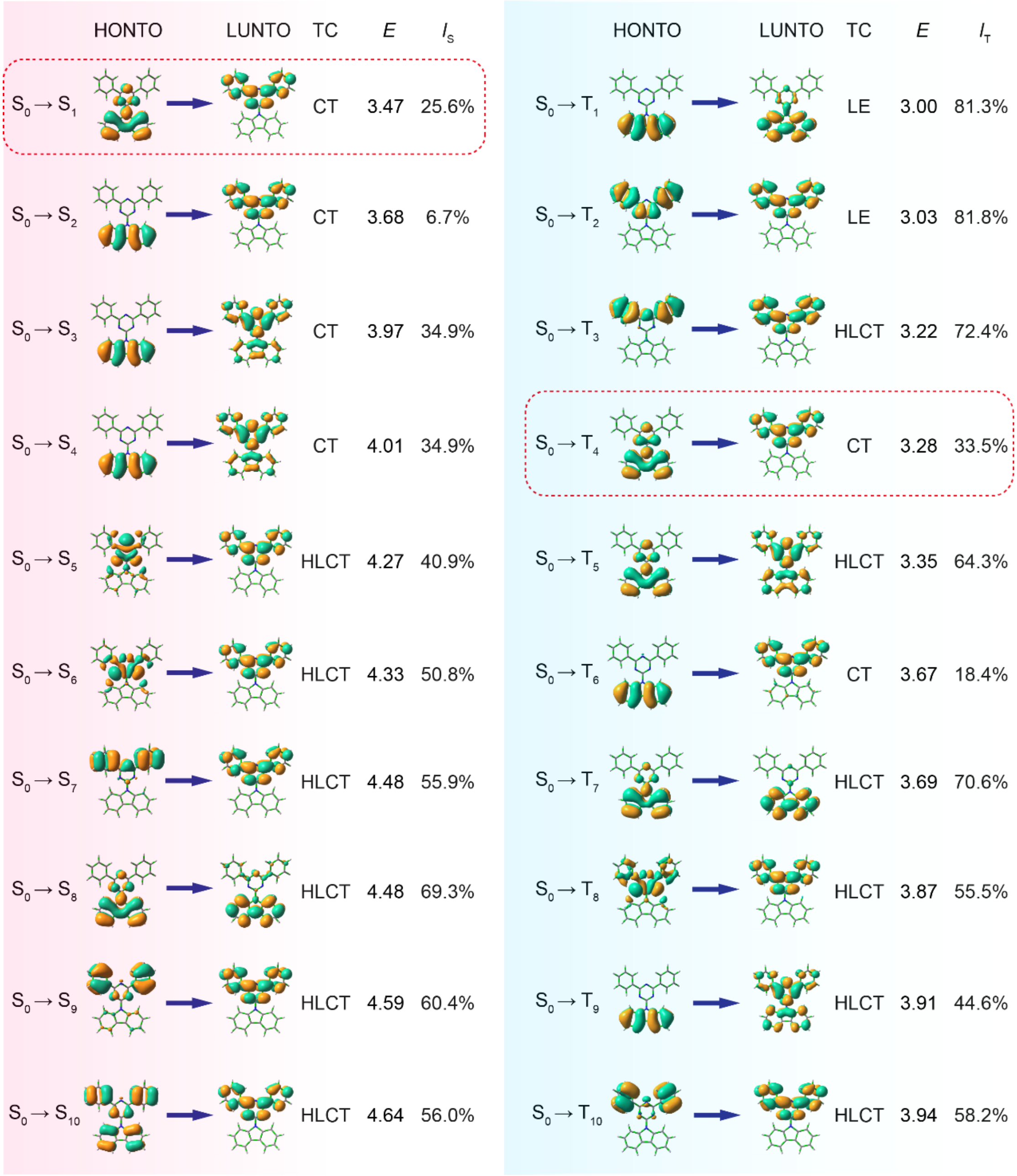

Figure S6. Excitation energy (*E*, in eV), HONTO and LUNTO distribution, HONTO-LUNTO overlap extent, and transition character (TC) of the singlet (left, S_1~S_{10}) and triplet (right, T_1~T_{10}) excited states of **DPhCzT**.

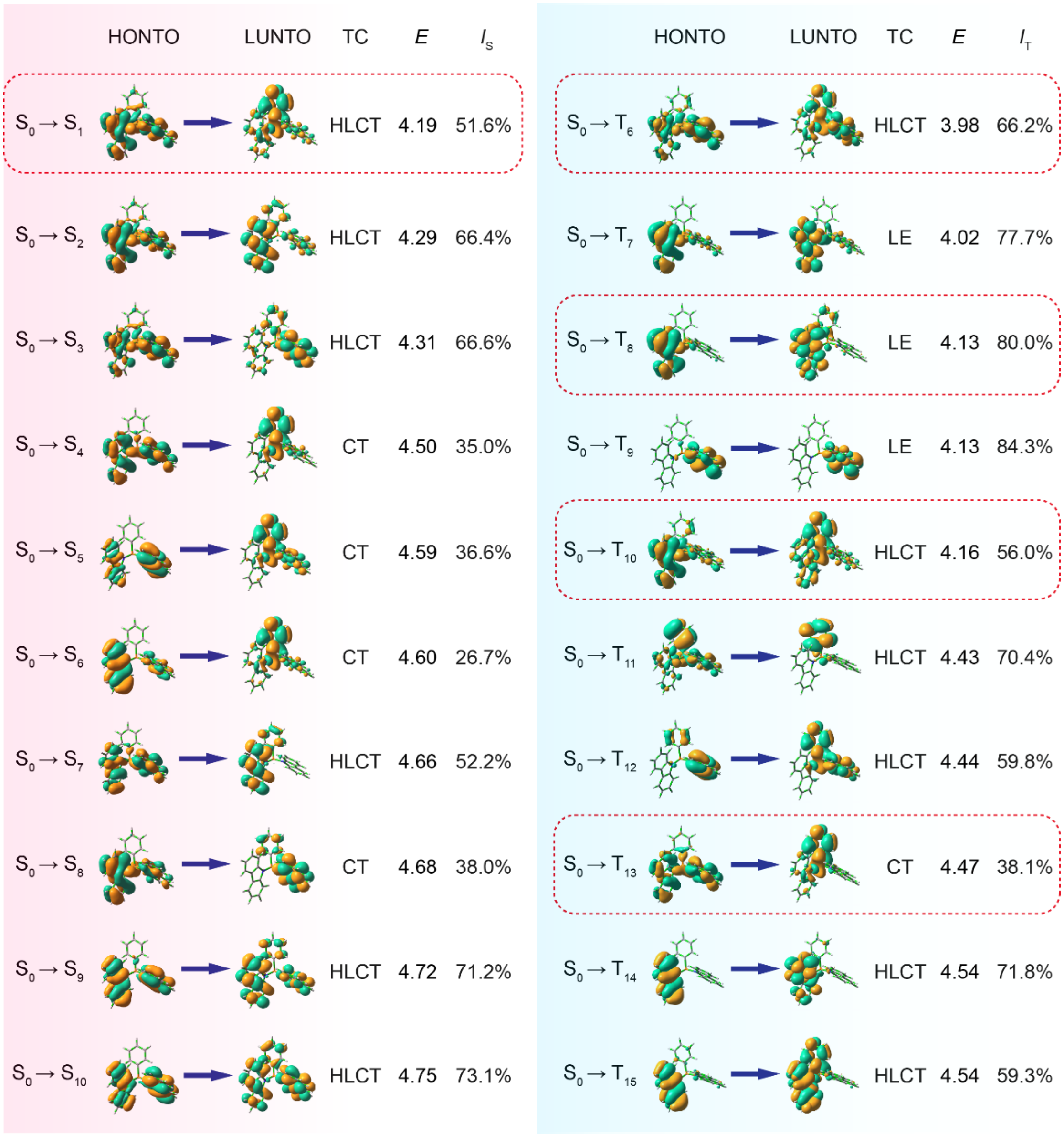

Figure S7. Excitation energy (*E*, in eV), HONTO and LUNTO distribution, HONTO-LUNTO overlap extent, and transition character (TC) of the singlet (left, S_1~S_{10}) and triplet (right, T_6~T_{15}) excited states of **DCzPhP**.

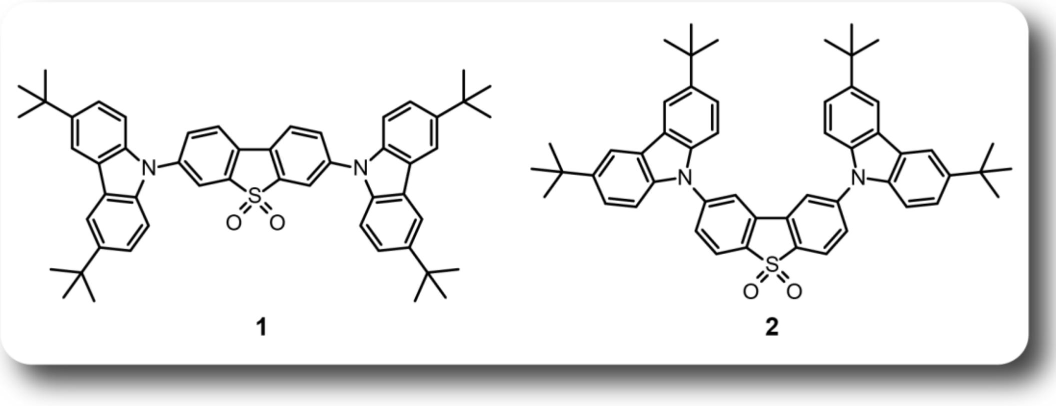

Figure S8. Chemical structures of Compounds **1** and **2**.

7. Supplementary tables

Table S1. Calculated vertical excitation energies (E_{Sn} and E_{Tn}, in eV) and S_1-T_1 splitting (ΔE_{ST}[11], in eV) of the excited singlet and triplet states using various functionals at 6–31G(d) basis set of **DMAC-DPS**, **Spiro-CN**, **TAP-NZP,** and **DPhCzT** in comparison with their experimental results (Exp.).

Functional	**DMAC-DPS**[20]			**Spiro-CN**[21]			**TAP-NZP**[22]			**DPhCzT**[23]		
	E_{S1}	E_{T1}	ΔE_{ST}[11]	E_{S1}	E_{T1}	ΔE_{ST}[11]	E_{S1}	E_{S3}	E_{S4}	E_{S1}	E_{T1}	ΔE_{ST}[11]
B3LYP	2.76	2.75	0.01	2.04	2.03	0.01	1.93	3.45	3.56	3.28	3.11	0.17
PBE0	2.93	2.91	0.02	2.22	2.21	0.01	2.08	3.62	3.75	3.46	3.00	0.46
BMK	3.42	3.40	0.02	2.78	2.71	0.07	2.42	4.01	4.06	3.94	3.38	0.56
M062X	3.63	3.62	0.01	3.18	2.89	0.29	2.54	3.99	4.11	4.19	3.59	0.60
ωB97XD	3.88	3.31	0.57	3.54	2.62	0.92	2.63	4.03	4.27	4.38	3.15	1.23
M06HF	4.28	4.04	0.24	4.02	3.25	0.77	3.03	4.17	4.32	3.95	4.79	0.84
Exp.	2.99	2.91	0.08	2.44	2.38	0.06	2.48	3.42	4.01	3.30	2.96	0.34

Table S2. TD-DFT calculated singlet (S_n) and triplet (T_n) excited state transition configurations of **DMAC-DPS**. The matched excited states that have the close excitation energy ($|\Delta E_{ST}| < 0.37$ eV, highlighted in blue) and contain the same orbital transition components of S_1 were highlighted in red.

S_n/T_n	Energy (eV)	Transition Configuration
**S_1**	2.9254	H-1→L+2(6.44%), H→L(91.08%)
**S_2**	2.9260	H-1→L(91.05%), H→L+2(6.46%)
**S_3**	3.6188	H-1→L+3(25.6%), H→L+1(69.55%), H→L+2(3.75%)
**S_4**	3.6194	H-1→L+1(69.41%), H-1→L+2(3.75%), H→L+3(25.74%)
**S_5**	3.9030	H-1→L(8.01%), H-1→L+3(3.51%), H→L+1(8.84%), H→L+2(79.05%)
**S_6**	3.9037	H-1→L+1(8.91%), H-1→L+2(79%), H→L(7.98%), H→L+3(3.53%)
**S_7**	4.1622	H-1→L+1(19.41%), H-1→L+2(9.96%), H→L+3(70.01%)
**S_8**	4.1625	H-1→L+3(70.16%), H→L+1(19.32%), H→L+2(9.88%)
**S_9**	4.3608	H-4→L+6(2.01%), H-1→L+4(46.74%), H→L+5(46.9%)
**S_{10}**	4.3627	H-5→L+6(2.04%), H-4→L+7(2.01%), H-1→L+5(46.65%), H→L+4(46.8%)
**T_1**	2.9103	H-1→L+2(6.84%), H→L(90.41%)
**T_2**	2.9108	H-1→L(90.32%), H→L+2(6.85%)
**T_3**	3.1277	H-7→L+11(3%), H-6→L+10(3%), H-5→L+4(4.49%), H-4→L+5(4.55%), H-3→L+9(3.83%), H-2→L+8(4.16%), H-1→L+3(4.73%), H-1→L+7(29.72%), H→L+1(4.16%), H→L+6(30.69%)
**T_4**	3.1277	H-7→L+10(3%), H-6→L+11(3.01%), H-5→L+5(4.5%), H-4→L+4(4.54%), H-3→L+8(4.17%), H-2→L+9(3.83%), H-1→L+1(4.14%), H-1→L+6(30.68%), H→L+3(4.74%), H→L+7(29.74%)
**T_5**	3.3813	H-11→L+1(2.91%), H-11→L+2(12.9%), H-10→L+3(13.3%) H-9→L+1(13.84%), H-9→L+2(2.56%), H-8→L(49.29%)
**T_6**	3.5220	H-11→L(27.82%), H-10→L+1(15.95%), H-10→L+2(3.38%) H-9→L+3(16.94%), H-8→L+1(6.32%), H-8→L+2(20.81%)
**T_7**	3.5505	H-1→L+3(23.83%), H-1→L+7(2.85%), H-1→L+9(3.48%) H→L+1(53.58%), H→L+2(3.95%), H→L+6(2.28%), H→L+8(3.96%)
**T_8**	3.5509	H-1→L+1(53.41%), H-1→L+2(3.99%), H-1→L+6(2.27%) H-1→L+8(3.98%), H→L+3(23.98%), H→L+7(2.85%), H→L+9(3.5%)
**T_9**	3.5509	H-7→L+7(7.34%), H-7→L+9(2.77%), H-6→L+6(7.16%) H-6→L+8(2.91%), H-5→L+9(6.34%), H-4→L+6(2.06%) H-4→L+8(6.48%), H-3→L+4(12.64%), H-2→L+5(12.64%) H-1→L+4(3.98%), H-1→L+11(11.74%), H→L+5(3.99%) H→L+10(11.76%)
**T_{10}**	3.5736	H-7→L+6(6.96%), H-7→L+8(3.00%), H-6→L+7(7.18%), H-6→L+9(2.70%), H-5→L+6(2.11%), H-5→L+8(6.35%), H-4→L+9(6.36%), H-3→L+5(12.4%), H-2→L+4(12.4%), H-1→L+5(3.85%), H-1→L+10(11.57%), H→L+4(3.86%),, H→L+11(11.57%)

Table S3. TD-DFT calculated singlet (S_n) and triplet (T_n) excited state transition configurations of **Spiro-CN**. The matched excited states that have the close excitation energy ($|\Delta E_{ST}| < 0.37$ eV, highlighted in blue) and contain the same orbital transition components of S_1 were highlighted in red.

S_n/T_n	Energy (eV)	Transition Configuration
**S_1**	2.2175	H→L(99.68%)
**S_2**	2.7638	H-1→L(99.75%)
**S_3**	3.2664	H→L+1(60.4%), H→L+2(37.03%)
**S_4**	3.2978	H→L+1(38.27%), H→L+2(60.74%)
**S_5**	3.5350	H→L+4(93.81%)
**S_6**	3.7058	H-1→L+4(4.48%), H→L+3(86.09%), H→L+5(3.26%), H→L+6(2.5%)
**S_7**	3.8013	H-10→L(2.05%), H-2→L(93.99%)
**S_8**	3.8141	H-1→L+1(41.1%), H-1→L+2(54.33%)
**S_9**	3.8554	H-1→L+1(57.03%), H-1→L+2(39.24%)
**S_{10}**	3.8990	H-1→L+2(2.66%), H-1→L+4(24.13%), H→L+3(10.14%) H→L+5(36.81%), H→L+6(18.68%), H→L+8(3.63%)
**T_1**	2.2125	H→L(98.8%)
**T_2**	2.4003	H-2→L+2(3.18%), H-1→L+10(2.37%), H→L+1(22.07%), H→L+2(57.99%)
**T_3**	2.5676	H-15→L+3(3.73%), H-14→L+1(2.58%), H-10→L(3.92%) H-9→L(2.57%), H-5→L(8.37%), H-3→L(40.65%), H-2→L(26.77%)
**T_4**	2.7627	H-1→L(99.71%)
**T_5**	3.0705	H-7→L+9(2.7%), H-5→L+14(2.26%), H-4→L+10(2.68%) H-2→L+7(2.48%), H-1→L+6(9.21%), H-1→L+8(16.85%) H→L+4(7.85%), H→L+7(30.77%), H→L+8(2.22%)
**T_6**	3.0745	H-7→L+6(2.63%), H-6→L+9(3.31%), H-5→L+10(2.62%) H-4→L+14(2.48%), H-2→L+8(2.67%), H-1→L+7(28.19%) H→L+6(10.24%), H→L+7(4.28%), H→L+8(24.12%)
**T_7**	3.0872	H-4→L+7(2.39%), H-1→L+1(13.74%), H-1→L+2(37.11%) H-1→L+14(2.19%), H→L+3(2.43%), H→L+10(18.94%)
**T_8**	3.1176	H-1→L+8(5.04%), H→L+4(77.49%), H→L+7(3.85%)
**T_9**	3.2841	H→L+1(71.01%), H→L+2(26.34%)
**T_{10}**	3.3865	H-1→L+4(23.72%), H→L+3(24.67%), H→L+5(11.99%) H→L+6(14.6%), H→L+8(8.59%), H→L+13(4.37%)

Table S4. TD-DFT calculated singlet (S_n) and triplet (T_n) excited state transition configurations of **MADa**. The matched excited states that have the close excitation energy ($|\Delta E_{ST}| < 0.37$ eV, highlighted in blue) and contain the same orbital transition components of S_1 and S_2 were highlighted in green and red, respectively.

S_n/T_n	Energy (eV)	Transition Configuration
S_1	3.17	H→L(96.79%)
S_2	3.70	H-1→L(96.09%)
S_3	3.99	H-4→L(41.38%), H-1→L+1(2.10%), H-1→L+3(8.10%) H→L+1(8.39%), H→L+3(29.88%), H→L+4(2.22%)
S_4	4.58	H-1→L+3(13.26%), H-1→L+4(5.45%), H→L+3(25.34%) H→L+4(32.35%), H→L+7(9.60%)
S_5	4.68	H-1→L+1(8.85%), H-1→L+3(3.86%), H→L+1(75.58%)
S_6	4.82	H-2→L(14.31%), H-1→L+4(6.65%), H-1→L+7(7.76%) H→L+4(25.41%), H→L+7(33.56%)
S_7	4.84	H-10→L(4.12%), H-2→L(69.22%), H-1→L+7(2.58%), H→L+7(11.83%)
S_8	4.91	H-1→L+2(15.58%), H→L+2(72.34%), H→L+10(6.77%)
S_9	4.99	H-12→L+2(3.34%), H-11→L+1(7.92%), H-3→L+1(36.19%) H-2→L+2(35.43%), H-2→L+10(5.35%)
S_{10}	5.01	H-4→L(14.33%), H-1→L+1(11.80%), H-1→L+3(4.92%), H-1→L+4(3.19%) H-1→L+7(3.51%), H→L+3(25.75%), H→L+4(14.29%), H→L+7(10.20%)
T_1	2.05	H-1→L(28.13%), H→L(68.18%)
T_2	3.36	H-1→L(64.54%), H→L(27.58%)
T_3	3.69	H-10→L(13.55%), H-10→L+11(2.22%), H-6→L(12.76%) H-1→L+4(8.39%), H-1→L+9(10.51%), H→L+3(14.75%), H→L+4(20.42%)
T_4	3.70	H-10→L(18.28%), H-6→L(6.65%), H-6→L+1(12.26%), H-1→L+4(15.84%) H-1→L+9(5.28%), H→L+3(12.60%), H→L+4(7.45%), H→L+9(12.08%)
T_5	3.71	H-11→L+10(4.60%), H-3→L+2(22.71%), H-2→L+1(59.40%)
T_6	3.72	H-6→L(4.68%), H-4→L(62.96%), H-1→L+3(6.91%), H→L+3(4.11%) H→L+4(10.03%)
T_7	3.83	H-4→L(22.79%), H-1→L+3(16.92%), H→L+1(8.16%), H→L+3(29.51%) H→L+4(12.61%)
T_8	4.06	H-9→L+5(6.26%), H-8→L+6(6.23%), H-3→L+1(37.50%), H-2→L+2(33.33%)
T_9	4.17	H-6→L+8(5.79%), H-1→L+3(7.77%), H-1→L+7(21.22%), H→L+7(52.96%)
T_{10}	4.24	H-10→L+4(3.69%), H-7→L+4(6.13%), H-6→L+4(6.86%), H-6→L+7(7.25%) H-5→L+8(8.15%), H-1→L+11(15.41%), H→L+11(27.06%), H→L+13(3.64%)

Table S5. TD-DFT calculated singlet (S_n) and triplet (T_n) excited state transition configurations of **TPA-NZP**. The matched excited states that have the close excitation energy ($|\Delta E_{ST}| < 0.37$ eV, highlighted in blue) and contain the same orbital transition components of S_2 was highlighted in red.

S_n/T_n	Energy (eV)	Transition Configuration
S_1	2.58	H-1→L(21.79%), H→L(77.22%)
S_2	3.24	H-1→L(74.90%), H→L(19.63%)
S_3	4.05	H-5→L(29.62%), H-4→L(11.72%), H-3→L(13.24%), H-2→L(19.51%), H-1→L+6(5.07%)
S_4	4.17	H-13→L(3.02%), H-12→L(15.83%), H-11→L(13.25%), H-7→L(2.63%), H-6→L(3.97%), H-5→L(9.90%), H-3→L(37.06%), H→L+1(3.27%)
S_5	4.36	H-6→L(2.33%), H-5→L(2.13%), H-1→L+3(2.02%), H-1→L+4(2.65%), H→L+1(70.35%), H→L+2(3.03%), H→L+3(3.09%), H→L+4(2.88%), H→L+6(2.22%)
S_6	4.44	H-6→L(3.20%), H-5→L(3.24%), H-2→L(2.88%), H-1→L+2(8.83%), H→L+2(61.33%)
S_7	4.51	H-13→L(4.80%), H-12→L(4.33%), H-11→L(8.71%), H-6→L(54.33%), H-3→L(3.37%), H-2→L(5.37%), H→L+2(2.56%)
S_8	4.55	H-1→L+3(10.78%), H-1→L+4(8.20%), H→L+3(43.12%), H→L+4(30.14%)
S_9	4.62	H-12→L(3.25%), H-7→L(7.69%), H-6→L(16.91%), H-5→L(31.65%), H-4→L(14.78%), H-2→L(9.31%)
S_{10}	4.69	H-12→L(5.94%), H-11→L(7.76%), H-10→L(8.91%), H-8→L(5.12%), H-6→L(4.90%), H-4→L(3.29%), H-3→L(18.07%), H-2→L(7.31%), H-1→L+1(7.27%), H-1→L+2(3.06%), H→L+4(5.11%)
T_1	1.32	H-10→L(3.03%), H-1→L(51.45%), H→L(46.94%), H-1<-L(3.50%), H<-L(2.25%)
T_2	2.98	H-8→L(2.19%), H-7→L(2.71%), H-1→L(34.42%), H→L(42.61%), H→L+1(6.24%)
T_3	3.08	H-14→L(2.09%), H-5→L(42.37%), H-4→L(12.30%), H-3→L(12.68%), H-2→L(21.41%)
T_4	3.31	H-15→L(5.95%), H-10→L(29.84%), H-1→L+1(10.73%), H-1→L+2(2.15%), H-1→L+3(4.29%), H-1→L+4(3.83%), H-1→L+5(5.97%), H→L+1(10.10%) H→L+3(2.25%), H→L+5(3.62%)
T_5	3.63	H-1→L(3.06%), H-1→L+1(4.71%), H→L(6.09%), H→L+1(37.13%), H→L+2(4.90%), H→L+3(7.23%), H→L+4(7.34%), H→L+6(3.13%)
T_6	3.68	H-9→L+11(3.34%), H-1→L+3(14.32%), H-1→L+4(7.31%), H→L+2(4.68%), H→L+3(34.52%), H→L+4(16.78%)
T_7	3.76	H-13→L(3.69%), H-12→L(18.02%), H-12→L+1(2.92%), H-11→L(14.36%) H-11→L+1(2.29%), H-6→L(4.49%), H-5→L(5.10%), H-3→L(22.05%), H-1→L(2.97%), H→L+1(2.15%)
T_8	3.95	H-15→L(2.61%), H-14→L(2.85%), H-13→L(2.96%), H-12→L(7.93%), H-11→L(8.58%), H-10→L(3.21%), H-6→L(9.74%), H-3→L(2.19%), H-1→L+1(5.97%), H-1→L+5(8.63%), H→L+4(4.86%), H→L+5(4.64%)

**T_9**	4.01	H-9→L+8(3.21%), H-1→L+2(11.87%), H→L+2(55.34%), H→L+4(10.94%)
**T_{10}**	4.08	H-14→L(3.87%), H-1→L+1(7.49%), H-1→L+3(3.75%), H-1→L+4(8.29%), H-1→L+6(12.39%), H-1→L+7(5.49%), H→L+5(2.70%), H→L+6(17.12%), H→L+7(5.24%), H→L+11(2.42%)

Table S6. TD-DFT calculated singlet (S_n) and triplet (T_n) excited state transition configurations of **DPhCzT**. The matched excited states that have the close excitation energy ($|\Delta E_{ST}| < 0.37$ eV, highlighted in blue) and contain the same orbital transition components of S_1 were highlighted in red.

S_n/T_n	Energy (eV)	Transition Configuration
**S_1**	3.47	H→L(98.60%)
**S_2**	3.68	H-1→L(99.56%)
**S_3**	3.97	H→L+1(94.67%)
**S_4**	4.01	H-1→L+1(97.78%)
**S_5**	4.27	H-8→L(80.72%), H-5→L(3.33%), H-4→L(12.97%)
**S_6**	4.33	H-9→L(2.94%), H-7→L(87.99%), H-6→L(5.23%)
**S_7**	4.48	H-6→L(4.31%), H-3→L(81.96%), H-2→L+1(7.51%)
**S_8**	4.48	H-1→L+6(9.68%), H→L+2(85.22%)
**S_9**	4.59	H-6→L+1(2.02%), H-5→L(58.42%), H-2→L(30.52%)
**S_{10}**	4.64	H-6→L+1(4.09%), H-5→L(22.08%), H-4→L(6.90%), H-2→L(59.56%)
**T_1**	3.00	H-9→L+9(4.14%), H-4→L+2(2.67%), H-1→L+1(14.86%) H-1→L+2(58.56%), H→L+6(11.63%)
**T_2**	3.03	H-6→L+3(5.02%), H-5→L+4(6.35%), H-4→L(7.61%), H-3→L+1(18.00%) H-3→L+3(2.06%), H-3→L+5(2.90%), H-2→L(43.80%), H→L+1(6.18%)
**T_3**	3.22	H-6→L(4.54%), H-6→L+4(8.32%), H-5→L+3(3.10%), H-5→L+5(5.79%) H-4→L+1(2.54%), H-3→L(43.94%), H-3→L+7(3.03%) H-2→L+1(13.67%), H-2→L+3(5.61%)
**T_4**	3.28	H-10→L(2.41%), H→L(93.94%)
**T_5**	3.35	H-4→L+6(4.19%), H-2→L(4.34%), H→L+1(70.15%), H→L+3(2.61%) H→L+5(4.95%)
**T_6**	3.67	H-1→L(63.74%), H→L+1(3.81%), H→L+2(23.64%)
**T_7**	3.69	H-9→L+2(2.01%), H-4→L+6(3.66%), H-1→L(35.15%) H→L+1(4.10%), H→L+2(47.57%)
**T_8**	3.87	H-9→L(2.48%), H-7→L(71.40%), H-6→L(15.20%), H-5→L+1(2.66%)
**T_9**	3.91	H-4→L+22.58%), H-1→L+1(72.39%), H-1→L+2(11.35%), H-1→L+3(2.68%), H-1→L+5(3.91%)
**T_{10}**	3.94	H-6→L+1(15.15%), H-6→L+3(3.38%), H-5→L(63.60%), H-3→L+1(5.75%) H-2→L(3.08%)

Table S7. TD-DFT calculated singlet (S_n) and triplet (T_n) excited state transition configurations of **DCzPhP**. The matched excited states that have the close excitation energy ($|\Delta E_{ST}| < 0.37$ eV, highlighted in blue) and contain the same orbital transition components of S_1 were highlighted in red.

S_n/T_n	Energy (eV)	Transition Configuration
S_1	4.19	H→L(96.45%)
S_2	4.29	H-1→L+2(2.24%), H→L+1(77.42%)
S_3	4.31	H-3→L+1(3.60%), H-3→L+2(2.55%), H-2→L+5(2.41%), H-1→L+1(2.64%), H→L+2(77.71%)
S_4	4.50	H-3→L(27.36%), H-2→L(12.95%), H-1→L(52.39%), H-1→L+2(2.22%), H→L+1(2.07%)
S_5	4.59	H-2→L(74.30%), H-1→L(15.10%), H-1→L+1(2.05%)
S_6	4.60	H-3→L(65.62%), H-2→L(3.91%), H-1→L(25.68%)
S_7	4.66	H-3→L+1(2.36%), H-2→L+1(16.82%), H-2→L+2(3.97%), H-1→L+1(38.09%), H-1→L+2(11.25%), H→L+1(8.42%), H→L+2(2.24%), H→L+3(5.58%), H→L+4(2.18%)
S_8	4.68	H-3→L+1(3.76%), H-3→L+2(24.02%), H-2→L(2.63%), H-2→L+1(2.65%) H-1→L+1(10.31%), H-1→L+2(28.94%), H→L+1(3.10%), H→L+2(9.92%) H→L+3(6.31%)
S_9	4.72	H-3→L+1(23.35%), H-3→L+2(12.40%), H-2→L+1(23.20%), H-2→L+2(8.55%), H-1→L+1(2.97%), H-1→L+2(8.41%), H-1→L+4(2.04%), H→L+3(2.75%), H→L+6(4.47%)
S_{10}	4.75	H-3→L+1(39.75%), H-2→L+2(27.75%), H-1→L+1(3.52%),, H-1→L+2(4.93%), H→L+4(4.37%), H→L+5(4.45%)
T_6	3.98	H-7→L+3(3.97%), H-6→L+6(2.48%), H-3→L+2(3.23%), H-1→L+5(4.43%) H→L(27.40%), H→L+1(4.32%), H→L+2(12.38%), H→L+7(10.85%)
T_7	4.02	H-9→L+1(5.05%), H-5→L+6(4.77%), H-3→L+4(3.92%), H-1→L+8(3.45%) H→L+1(4.00%), H→L+2(10.98%), H→L+4(12.45%), H→L+5(7.52%)
T_8	4.13	H-5→L+1(6.64%), H-3→L+1(5.38%), H-3→L+4(3.78%), H-3→L+6(6.02%) H-2→L+2(6.98%), H-1→L+1(7.44%), H-1→L+6(6.23%), H→L(3.06%) H→L+4(16.35%), H→L+5(6.87%)
T_9	4.13	H-6→L+1(3.30%), H-3→L+4(5.87%), H-3→L+5(4.88%), H-2→L+1(7.25%) H-2→L+2(4.46%), H-1→L+2(10.33%), H-1→L+5(3.32%), H→L+5(7.95%) H→L+6(15.81%)
T_{10}	4.16	H-7→L+3(6.74%), H-1→L(5.54%), H-1→L+5(4.27%), H→L(29.49%) H→L+1(8.36%)
T_{11}	4.43	H-7→L(18.35%), H-7→L+3(4.77%), H-4→L(3.20%), H-4→L+3(13.19%) H-2→L(5.03%), H→L+1(3.76%), H→L+3(21.49%)
T_{12}	4.44	H-7→L(4.85%), H-6→L+2(6.07%), H-5→L+2(4.16%), H-4→L+3(3.17%) H-2→L(23.02%), H-2→L+5(2.67%), H-2→L+7(7.08%), H-1→L(20.42%) H-1→L+2(3.01%), H-1→L+7(3.06%)
T_{13}	4.47	H-8→L+2(2.48%), H-3→L(22.12%), H-2→L(20.36%), H-1→L(29.13%) H→L(2.71%)
T_{14}	4.54	H-9→L+1(5.20%), H-3→L+4(21.04%), H-3→L+6(6.71%),

		H-2→L+4(13.14%), H-2→L+6(5.40%), H-1→L+4(9.69%), H-1→L+6(4.92%)
**T_{15}**	4.54	H-6→L+1(4.32%), H-5→L+1(7.05%), H-3→L(21.98%), H-3→L+1(3.73%) H-2→L(3.91%), H-2→L+5(3.72%), H-2→L+6(4.11%), H-1→L+5(3.12%) H-1→L+6(4.00%)

Table S8. Orbital similarity of HONTO (s_H) and LUNTO (s_L) between S_m and T_n for exciton transformation in TADF ($S_m \leftrightarrow T_n$), HLCT ($T_n \rightarrow S_m$), and OURTP ($S_1 \rightarrow T_n$) molecules. The matched excited states that have the close energy gap ($|\Delta E_{ST}| < 0.37$ eV) and the high orbital similarity were highlighted in red.

Compounds	S_m-T_n	ΔE_{ST} (eV)	s_H	s_L
**DMAC-DPS**	$S_1 \leftrightarrow T_1$	0.02	0.979	0.989
	$S_1 \leftrightarrow T_2$	0.02	0.992	0.996
	$S_1 \leftrightarrow T_3$	0.20	0.514	0.062
	$S_1 \leftrightarrow T_4$	0.20	0.486	0.061
	$S_2 \leftrightarrow T_1$	0.02	0.992	0.996
	$S_2 \leftrightarrow T_2$	0.02	0.979	0.989
	$S_2 \leftrightarrow T_3$	0.20	0.486	0.061
	$S_2 \leftrightarrow T_4$	0.20	0.514	0.062
**Spiro-CN**	$S_1 \leftrightarrow T_1$	0.01	0.985	0.995
	$S_1 \leftrightarrow T_2$	0.18	0.772	0.025
	$S_1 \leftrightarrow T_3$	0.35	0.032	0.940
**MADa**	$T_2 \rightarrow S_1$	0.19	0.718	0.953
	$T_2 \rightarrow S_2$	0.34	0.468	0.926
	$T_3 \rightarrow S_2$	0.01	0.584	0.209
	$T_4 \rightarrow S_2$	0.00	0.723	0.321
	$T_5 \rightarrow S_2$	0.01	0.206	0.971
	$T_6 \rightarrow S_2$	0.02	0.718	0.337
	$T_7 \rightarrow S_2$	0.13	0.718	0.337
	$T_8 \rightarrow S_2$	0.36	0.012	0.012
**TPA-NZP**	$T_2 \rightarrow S_2$	0.26	0.781	0.856
	$T_3 \rightarrow S_2$	0.16	0.088	0.875
	$T_4 \rightarrow S_2$	0.07	0.291	0.488
**DPhCzT**	$S_1 \rightarrow T_3$	0.25	0.067	0.727
	$S_1 \rightarrow T_4$	0.19	0.915	0.976
	$S_1 \rightarrow T_5$	0.12	0.963	0.335
	$S_1 \rightarrow T_6$	0.20	0.346	0.974
	$S_1 \rightarrow T_7$	0.22	0.812	0.026
**DCzPhP**	$S_1 \rightarrow T_6$	0.21	0.670	0.549
	$S_1 \rightarrow T_7$	0.17	0.590	0.208
	$S_1 \rightarrow T_8$	0.06	0.550	0.175
	$S_1 \rightarrow T_9$	0.06	0.377	0.087
	$S_1 \rightarrow T_{10}$	0.03	0.703	0.866
	$S_1 \rightarrow T_{11}$	0.24	0.420	0.403
	$S_1 \rightarrow T_{12}$	0.25	0.223	0.706
	$S_1 \rightarrow T_{13}$	0.28	0.703	0.899
	$S_1 \rightarrow T_{14}$	0.35	0.215	0.124
	$S_1 \rightarrow T_{15}$	0.35	0.270	0.642

Table S9. Spin-orbit coupling (SOC) matrix elements of $S_1 \rightarrow T_n$ transitions in **DPhCzT** calculated by B3LYP/cc-pVTZ using Dalton package. The matched excited states that have the close energy gap ($|\Delta E_{ST}| < 0.37$ eV) and the high SOC matrix elements (SOC > 0.3 cm^{-1}) were highlighted in red.

**DPhCzT**	**x (hartree)**	**y (hartree)**	**z (hartree)**	**SOC (cm^{-1})**
$S_1 \rightarrow T_1$	3.7E-07	0	0	0.081
$S_1 \rightarrow T_2$	0	-5.0E-07	-1.3E-06	0.309
$S_1 \rightarrow T_3$	0	-5.0E-06	3.0E-07	0.994
$S_1 \rightarrow T_4$	0	-4.0E-06	-1.7E-07	0.903
$S_1 \rightarrow T_5$	-1.4E-06	0	0	0.298
$S_1 \rightarrow T_6$	4.4E-07	0	0	0.097
$S_1 \rightarrow T_7$	0	-1.0E-06	1.6E-06	0.468
$S_1 \rightarrow T_8$	0	-1.0E-05	-1.2E-05	3.519
$S_1 \rightarrow T_9$	8.0E-08	0	0	0.018
$S_1 \rightarrow T_{10}$	0	2.9E-07	2.9E-07	0.090

Table S10. The experimental data of Compounds **1** and **2** reported in the literature.[24]

Comp.	$\Phi_f \pm 0.05$	$\tau_f \pm 0.05$ (ns)	$\Phi_T \pm 0.1$	k_f x10^8 (s^{-1})	k_{ISC} x10^8 (s^{-1})	k_{IC} x10^8 (s^{-1})
**1**	0.94	3.08	Very low	3.1	Very low	0.2
**2**	0.10	1.60	0.32	0.6	1.9	3.6

Φ_f and Φ_T are photoluminescent quantum yields of fluorescence and phosphorescence, respectively; τ_f is the lifetime of fluorescence; k_f, k_{ISC}, and k_{IC} are the rate constants of fluorescence, intersystem crossing, and internal conversion, respectively.

Table S11. TD-DFT calculated singlet (S_n) and triplet (T_n) excited state transition configurations of Compound **1**. The matched excited states that have the close excitation energy ($|\Delta E_{ST}|$ < 0.37 eV, highlighted in blue) and contain the same transition components of S_1 were highlighted in red.

S_n/T_n	Energy (eV)	Transition Configuration (%)
**S_1**	2.8636	H→L(98.81)
**S_2**	3.0730	H-1→L(98.76)
**S_3**	3.3212	H-2→L(99)
**S_4**	3.3392	H→L+1(95.77)
**S_5**	3.3665	H-3→L(98.86)
**S_6**	3.5110	H-1→L+1(94.33);H→L+4(4.28)
**S_7**	3.8392	H-2→L+1(97.32);H-2→L+4(2.29)
**S_8**	3.8797	H-3→L+1(97.39);H-3→L+4(2.19)
**S_9**	3.9730	H-3→L+6(4.67);H-1→L+2(24.59);H→L+2(67.76)
**S_{10}**	4.0005	H-2→L+7(4.99);H-1→L+3(29.25);H→L+3(62.66)
**T_1**	2.4366	H-6→L(13.79);H→L(78.89)
**T_2**	2.8558	H-1→L(93.6);H→L+5(2.47)
**T_3**	3.0919	H-6→L+1(5.56);H-1→L+4(2.87);H→L(2.92);H→L+1(84.3)
**T_4**	3.1063	H-7→L+10(3.28);H-5→L+3(3.77);H-2→L(20.18);H-2→L+3(57.06) H-1→L+7(4.96);H→L+7(4.33)
**T_5**	3.1153	H-4→L+2(4.9);H-3→L(12.08);H-3→L+2(54.89);H-1→L+2(3.18) H-1→L+6(5.23);H→L+2(3.45);H→L+6(5.96)
**T_6**	3.3309	H-6→L(2.07);H-4→L+6(2.61);H-3→L(18.31);H-2→L(4.55) H-1→L+1(6.07);H-1→L+2(23.81);H→L+2(34.16)
**T_7**	3.3336	H-2→L(73.42);H-2→L+3(15.73);H→L+2(2.3)
**T_8**	3.3576	H-3→L(8.26);H-1→L+1(63.02);H-1→L+3(5.27);H→L+3(6.8) H→L+4(7.98)
**T_9**	3.3727	H-3→L(41.05);H-3→L+2(9.23);H-1→L+2(3.02);H-1→L+3(16.02) H→L+2(3.02);H→L+3(19.59)
**T_{10}**	3.3759	H-3→L(16.38);H-3→L+2(5.78);H-1→L+1(13.68);H-1→L+2(3.9) H-1→L+3(20.73);H→L+2(8.98);H→L+3(20.6);H→L+4(2.83)

Table S12. TD-DFT calculated singlet (S_n) and triplet (T_n) excited state transition configurations of Compound **2**. The matched excited states that have the close excitation energy ($|\Delta E_{ST}|$ < 0.37 eV, highlighted in blue) and contain the same transition components of S_1 were highlighted in red.

S_n/T_n	Energy (eV)	Transition Configuration (%)
S_1	2.9755	H→L(98.6)
S_2	3.0475	H-1→L(97.99)
S_3	3.3756	H-2→L(99.43)
S_4	3.4181	H-3→L(98.43)
S_5	3.4341	H→L+1(94.07)
S_6	3.5263	H-1→L+1(95.29)
S_7	3.8624	H-2→L+1(97);H-2→L+4(2.09)
S_8	3.9052	H-3→L+1(96.92)
S_9	3.9966	H-3→L+5(4.5);H-1→L+2(20.98);H-1→L+3(2.71);H→L+2(44.67) H→L+3(22.41)
S_{10}	4.0261	H-2→L+7(5.25);H-1→L+2(6.39);H-1→L+3(38.17);H-1→L+4(11.69) H→L+2(9.43);H→L+3(15.65);H→L+4(10.75)
T_1	2.6458	H-6→L(17.13);H-4→L(2.76);H→L(67.45)
T_2	2.8358	H-7→L(2.05);H-1→L(88.39);H→L(2.49)
T_3	3.1251	H-9→L+10(3.61);H-5→L+4(2.32);H-2→L(7.71);H-2→L+2(11.51) H-2→L+3(42.81);H-2→L+4(12.75);H-1→L+7(6.77);H→L+7(3.16)
T_4	3.1265	H-10→L+11(3.16);H-4→L+2(2.68);H-3→L(4.31);H-3→L+2(45.86) H-3→L+3(12.45);H-1→L+5(3.31);H→L+2(3.39);H→L+5(7.03)
T_5	3.1441	H-8→L+1(6.99);H-6→L(20.89);H-4→L(4.27);H-1→L+4(2.28) H→L(21.53);H→L+1(29.94)
T_6	3.2620	H-7→L+1(2.86);H-1→L(2.99);H-1→L+1(62.97);H→L+1(7.16) H→L+3(3.76);H→L+4(9.95)
T_7	3.2986	H-6→L(20.61);H-6→L+1(2.79);H-4→L(3.65);H-1→L+1(10.78) H-1→L+4(6.06);H→L(3.36);H→L+1(38.21);H→L+2(2.24);H→L+3(2 .12)
T_8	3.3590	H-3→L(2.95);H-3→L+2(3.42);H-1→L+2(14.89);H-1→L+3(7.93) H→L+1(6.21);H→L+2(41.49);H→L+3(10.01)
T_9	3.3768	H-2→L(90.22);H-2→L+3(3.2);H-2→L+4(3.17)
T_{10}	3.3843	H-5→L+7(2.05);H-1→L+2(9.73);H-1→L+3(33.2);H-1→L+4(16.66) H→L+2(3.04);H→L+3(19.35);H→L+4(7.64)

References:

1. Koch, W. & Holthausen, M. C. *A Chemist Guide to Density Functional Theory*; Wiley-VCH, 2000.
2. Runge, E. & Gross, E. K. U. Density-Functional Theory for Time-Dependent Systems. *Phys. Rev. Lett.* **52**, 997-1000 (1984).
3. Frisch, M. J. *et al*. *Gaussian 09 (Revision A02)*. Gaussian, Inc.: Wallingford CT, 2009.
4. Chen, T. *et al*. Understanding the Control of Singlet-Triplet Splitting for Organic Exciton Manipulating: A Combined Theoretical and Experimental Approach. *Sci. Rep.* **5**,10923 (2015).
5. Becke, A. D. Density-functional thermochemistry. III. The role of exact exchange. *J. Chem. Phys.* **98**, 5648-5653 (1993).
6. Adamo, C., Scuseria, G. E. & Barone, V. Accurate excitation energies from time-dependent density functional theory: Assessing the PBE0 model. *J. Chem. Phys.* **111**, 2889-2899 (1999).
7. Boese, A. D. & Martin, J. M. L. Development of Novel Density Functionals for Thermochemical Kinetics. *J. Chem. Phys.* **121**, 3405-3416 (2004).
8. Zhao, Y. & Truhlar, D. G. The M06 suite of density functionals for main group thermochemistry, thermochemical kinetics, noncovalent interactions, excited states, and transition elements: two new functionals and systematic testing of four M06-class functionals and 12 other functionals. *Theor. Chem. Acc.* **120**, 215-241 (2008).
9. Zhao, Y. & Truhlar, D. G. Density functional for spectroscopy: No long-range self-interaction error, good performance for Rydberg and charge-transfer states, and better performance on average than B3LYP for ground states. *J. Phys. Chem. A*, **110**, 13126-13130 (2006).
10. Chai, J. D. & Head-Gordon, M. Long-range corrected hybrid density functionals with damped atom-atom dispersion corrections. *Phys. Chem. Chem. Phys.* **10**, 6615-6620 (2008).
11. Martin, R. L. Natural transition orbitals. *J. Chem. Phys.* **118**, 4775-4777 (2003).
12. Lu, T. & Chen, F. W. Multiwfn: A multifunctional wavefunction analyzer. *J. Comput. Chem.* **33**, 580-592 (2012).
13. Aidas, K. *et al*. The Dalton quantum chemistry program system. *Wires. Comput. Mol. Sci.* **4**, 269-284 (2014).
14. Zhao, W. *et al*. Rational Molecular Design for Achieving Persistent and Efficient Pure Organic Room-Temperature Phosphorescence. *Chem* **1**, 592-602 (2016).
15. Ziegler, T., Rauk, A. & Baerends, E. J. On the calculation of multiplet energies by the hartree-fock-slater method. *Theor. Chem. Acc.* **43**, 261-271 (1977).
16. Endo, A. *et al*. Thermally Activated Delayed Fluorescence from Sn4+-Porphyrin Complexes and Their Application to Organic Light-Emitting Diodes—A Novel Mechanism for Electroluminescence. *Adv. Mater.* **21**, 4802-4806 (2009).
17. Busmann, H. G., Staerk, H. & Weller, A. Solvent Influence on the Magnetic-Field Effect of Polyethylene-linked Photogenerated Radical Ion-pairs. *J. Chem. Phys.* **91**, 4098-4105 (1989).
18. Lu, T. *Multiwfn: a multifunctional wavefunction analyzer* version 3.3.3, 2013.
19. Le Bahers, T., Adamo, C. & Ciofini, I. A Qualitative Index of Spatial Extent in Charge-Transfer Excitations. *J. Chem. Theory Comput.* **7**, 2498-2506 (2011).
20. Zhang, Q. S. *et al*. Efficient blue organic light-emitting diodes employing thermally activated delayed fluorescence. *Nat. Photonics* **8**, 326-332 (2014).
21. Huang, S. P. *et al*. Computational Prediction for Singlet- and Triplet-Transition Energies of

Charge-Transfer Compounds. *J. Chem. Theory Comput.* **9**, 3872-3877 (2013).

22. Pan, Y. Y. *et al*. High Yields of Singlet Excitons in Organic Electroluminescence through Two Paths of Cold and Hot Excitons. *Adv. Opt. Mater.* **2**, 510-515 (2014).

23. An, Z. F. *et al*. Conjugated Asymmetric Donor-Substituted 1,3,5-Triazines: New Host Materials for Blue Phosphorescent Organic Light-Emitting Diodes. *Chemistry-A European Journal* **17**, 10871-10878 (2011).

24. Dias, F. B. *et al*. Triplet Harvesting with 100% Efficiency by Way of Thermally Activated Delayed Fluorescence in Charge Transfer OLED Emitters. *Adv. Mater.* **25**, 3707-3714 (2013).
